# Pathology-specific experimental antivenoms for haemotoxic snakebite: The impact of immunogen diversity on the *in vitro* cross-reactivity and *in vivo* neutralisation of geographically diverse snake venoms

**DOI:** 10.1371/journal.pntd.0009659

**Published:** 2021-08-18

**Authors:** Nessrin Alomran, Jaffer Alsolaiss, Laura-Oana Albulescu, Edouard Crittenden, Robert A. Harrison, Stuart Ainsworth, Nicholas R. Casewell

**Affiliations:** 1 Centre for Snakebite Research & Interventions, Liverpool School of Tropical Medicine, Pembroke Place, Liverpool, United Kingdom; 2 Centre for Drugs and Diagnostics, Liverpool School of Tropical Medicine, Pembroke Place, Liverpool, United Kingdom; Muséum National d’Histoire Naturelle, FRANCE

## Abstract

**Background:**

Snakebite is a neglected tropical disease that causes high global rates of mortality and morbidity. Although snakebite can cause a variety of pathologies in victims, haemotoxic effects are particularly common and are typically characterised by haemorrhage and/or venom-induced consumption coagulopathy. Antivenoms are the mainstay therapeutic for treating the toxic effects of snakebite, but despite saving thousands of lives annually, these therapies are associated with limited cross-snake species efficacy due to venom variation, which ultimately restricts their therapeutic utility to particular geographical regions.

**Methodology/Principal findings:**

In this study we explored the feasibility of generating globally effective pathology-specific antivenoms to counteract the haemotoxic signs of snakebite envenoming. Two different immunogen mixtures, consisting of seven and twelve haemotoxic venoms sourced from geographically diverse and/or medically important snakes, were used to raise ovine polyclonal antibodies, prior to characterisation of their immunological binding characteristics and *in vitro* neutralisation profiles against each of the venoms. Despite variability of the immunogen mixtures, both experimental antivenoms exhibited broadly comparable *in vitro* venom binding and neutralisation profiles against the individual venom immunogens in immunological and functional assays. However, *in vivo* assessments using a murine preclinical model of antivenom efficacy revealed substantial differences in venom neutralisation. The experimental antivenom generated from the seven venom immunogen mixture outperformed the comparator, by providing protective effects against venom lethality caused by seven of the eight geographically diverse venoms tested, including three distinct venoms that were not used as immunogens to generate this antivenom. These findings suggest that a core set of venom immunogens may be sufficient to stimulate antibodies capable of broadly neutralising a geographically diverse array of haemotoxic snake venoms, and that adding additional venom immunogens may impact negatively on the dose efficacy of the resulting antivenom.

**Conclusions/Significance:**

Although selection of appropriate immunogens that encapsulate venom toxin diversity without diluting antivenom potency remains challenging and further optimisation is required, the findings from this pilot study suggest that the generation of pathology-specific antivenoms with global utility is likely to feasible, thereby highlighting their promise as future modular treatments for the world’s tropical snakebite victims.

## Author summary

Snakebite is a major public health concern that causes extensive death and disability in the tropical world. Variation in venom composition causes major challenges for snakebite therapy by typically restricting antivenom efficacy to only the few snake species that each product was designed against, meaning that many geographically restricted therapeutics are manufactured for different parts of the world. In this study, we explored the feasibility of generating a global pathology-specific antivenom by using two different sets of haemotoxic venoms as immunogens, one including seven venoms and the other twelve venoms, to generate ovine polyclonal antibodies. Our *in vitro* immunological cross-reactivity and venom functional-neutralisation findings demonstrated broadly comparable venom recognition and inhibition against the various venom immunogens, irrespective of whether they were included in the immunising mixture. However, assessments of murine *in vivo* protection against venom-induced lethality revealed that the antivenom generated with fewer immunogens outperformed the comparator, and provided broader preclinical efficacy, even against some venoms not used to raise the antibodies. These findings suggest that careful selection of venoms used as immunogens is crucial for maintaining antivenom potency, and that a small subset of venoms may be capable of generating an antivenom with potential global efficacy against a specific pathology commonly observed after snakebite.

## Introduction

Snakebite envenoming is classified as a neglected tropical disease (NTD), and as such is most prevalent in tropical and subtropical regions of the world, particularly rural regions [1,2]. Of the estimated 5.4 million snakebites thought to occur annually, 1.8–2.7 million of these cases likely result in envenoming, with as many as 138,000 victims dying as the result of the snakebite [2,3].

Snakebites can result in myriad pathophysiological consequences in envenomed victims as a result of the composition of the aetiological toxins varying extensively among snake species [4,5]. Despite this variation, most venoms can be broadly classified as inflicting cytotoxic, neurotoxic and/or haemotoxic pathology. Envenoming by viperid snakes (true vipers, *Viperinae* and pit vipers, *Crotalinae*) primarily result in haemotoxic pathologies, typically characterised by local or systemic haemorrhage, including overt and internal bleeding [6]. Haemorrhage is frequently compounded by venom toxin-induced impairment of blood coagulation, which can result in venom-induced consumption coagulopathy (VICC), defined by untraceable or minimal fibrinogen levels following envenoming [7,8]. While vipers are predominately responsible for causing life-threatening systemic haemorrhage and VICC in the majority of snakebite cases, some African colubrids (e.g. *Dispholidus typus*), Asian natricines (e.g. *Rhabdophis subminiatus*) and Australasian elapids (e.g. *Oxyuranus scutellatus*) can also induce VICC as the result of potent procoagulant toxins in their venoms [7].

The venom toxins primarily thought to be responsible for causing VICC are members of the snake venom serine protease (SVSP) and snake venom metalloproteinase (SVMP) families [9,10]. Each of these toxin families is diverse, with multiple related isoforms produced in the venom gland of each snake species, and these often exhibit distinct functional activities [5,6]. Haemostatic targets for these toxins include Factor X and prothrombin, and their activation via cleavage stimulates initial procoagulant effects, followed by their consumption and that of downstream clotting factors, resulting in a loss of clotting capability [11,12]. Other toxins act directly on fibrinogen in a fibrinogenolytic manner [7], ultimately resulting in its depletion by this intensified dysregulated activation of the coagulation cascade [13]. In addition, many viperid venoms also simultaneously contain anticoagulant toxins, such as C-type lectins (CTLs), phospholipases A_2_ (PLA_2_s) and disintegrins, which act by various mechanisms, but also share a common mode of action by either inhibiting or promoting the aggregation (to depletion) of platelets [6]. This combination of both procoagulant and anticoagulant toxins in snake venom [14] can rapidly lead to a loss of clotting capability in an envenomed victim [7,15,16], and the severity of envenoming can be further compounded by SVMP toxins simultaneously disrupting the integrity of the microvasculature via cleavage of extracellular matrix proteins, resulting in widespread haemorrhage [17,18].

The only specific therapy for snakebite consists of animal-derived polyclonal antibodies, known as antivenom. Antivenom antibodies exhibit high specificity for venom toxins, enabling binding, blocking and signalling of toxins for clearance. The production of antivenom involves the repeated immunisation of equines or ovines with sub-toxic doses of crude venom(s), followed by the isolation of the resulting polyclonal immunoglobulin G (IgG) antibodies from the hyper-immune serum/plasma [19,20]. Although a number of antivenom manufacturers digest the IgG antibodies into F(ab’)_2_ or Fab fragments, others retain the intact IgG via caprylic acid precipitation [21]. This latter approach has been the basis of conventional IgG purification since the end of the 1980s [22] and involves the elimination of non-IgG proteins from the hyperimmune serum/plasma, leaving the IgG intact and thus enhancing antibody yield via a reduction in process losses [20,23].

Antivenoms are life-saving therapeutics, but they are also associated with a number of limitations. For instance, it has been demonstrated that only 10–20% of antivenom antibodies are typically specific to the venom immunogens [24], with the remainder thought to target environmental antigens animals are exposed to during immunisation [25,26], resulting in poor dose efficacy. Furthermore, due to their foreign (i.e. animal-derived) active components and requirement for intravenous (iv) delivery, antivenoms are associated with a high incidence of acute and chronic adverse reactions, ranging from mild (e.g. rashes) to severe (e.g. anaphylaxis) [27–31]. Antivenoms are also prohibitively expensive to the people in greatest need as treatment courses frequently necessitate the administration of multiple vials (ranging from 3–30 vials depending on product and region), resulting in costs to the patient often exceeding $1,000 USD in sub-Saharan Africa, for example, and therefore further exacerbate poverty [32]. Finally, and crucially, antivenoms typically exhibit limited cross-snake species efficacy, as the direct result of variation in venom composition among medically important snake species [5,33].

To attempt to mitigate against venom variation many antivenoms currently manufactured are denotated as polyvalent, where multiple different snake venoms are simultaneously used as immunogens for generating antibodies. Contrastingly, monovalent antivenoms are made using a single venom as an immunogen, and are typically manufactured for regions where a single medically important snake species is responsible for the majority of bites (e.g. *Echis ocellatus* in West Africa) or where a diagnostic test enables rational antivenom choice (currently only in Australia) [34]. Consequently, for many regions of the world, polyvalent antivenoms are the most commonly encountered therapeutic due to their capability to cross-react with and neutralise venoms from various medically important snakes found in a particular region, thus obviating the requirement for accurate diagnosis of the biting species [34,35]. Despite their paraspecificity, these products remain restricted for use in certain geographical regions (i.e. continental or sub-continental), meaning that many different polyvalent antivenom products are manufactured worldwide. However, polyvalent antivenoms are typically associated with a requirement to deliver higher therapeutic doses than monovalent products due to only a low proportion of their toxin specific IgGs being specific to the single venom injected during a snakebite [36], thus increasing both the potential risk of adverse reactions and the financial cost to the patient.

One of the major challenges associated with manufacturing a polyvalent antivenom for a specific geographical region is the great diversity of venom toxins found within the venoms of sympatric viperid and elapid snakes. For example, haemotoxic viper venoms are typically dominated by SVMP, SVSP, PLA_2_ and CTL toxin families, while elapid snake venoms, which are often neurotoxic, typically consist mostly of three finger toxins (3FTx), PLA_2_ (distinct from viper PLA_2_) and/or kunitz-type serine protease inhibitors [37]. Thus, attempting to generate neutralising antibodies against all of these diverse toxin families simultaneously presents a major challenge, and there are concerns that the larger, more immunogenic, venom toxins (e.g. SVMPs, SVSP) may dominate the immune response during immunisation, resulting in poor recognition of lower molecular weight elapid neurotoxins (e.g. 3FTx), and reduced antivenom efficacy against elapid envenoming [38]. In response to this concern, certain antivenom manufacturers (e.g. Thai Red Cross) now produce polyvalent antivenoms that are separately specific to haemotoxic vipers and neurotoxic elapids [39,40]. However, each of these antivenoms remains geographically restricted for use only in parts of South Asia. More recently, attempts to produce experimental neurotoxicity-specific antivenoms with broader geographical efficacy have been described, resulting in a diverse range of venoms being neutralised by antivenoms raised by immunisation with a diversity of venoms and specific venom toxin fractions [41,42]. A similar pathology-specific approach for counteracting haemotoxic envenoming has also been proposed as a potentially feasible strategy [11].

Consequently, in this study, we explored the feasibility of generating globally applicable, pathology-specific, polyvalent antivenoms for combatting snakebite haemotoxicity. To do so, we generated ovine polyclonal antibodies against two groups of venom immunogens, which contained seven potently coagulopathic venoms and twelve broadly haemotoxic venoms, respectively, both containing venoms sourced from a wide geographical distribution. We then assessed the specificity and inhibitory capability of the two resulting experimental IgG-based antivenoms using a range of *in vitro* immunological and venom neutralisation assays, before testing their preclinical efficacy in an *in vivo* model of envenoming. Despite differences in the number of venom immunogens utilised, our findings demonstrate that the inclusion of additional venom immunogens had a detrimental effect on antivenom efficacy *in vivo*, though they also suggest that the generation of generic, globally efficacious pathology-specific, antivenoms against haemotoxic envenoming is likely to be feasible, though further work focused on the identification of key venom/toxin immunogens is required to achieve this goal.

## Materials and methods

### Ethics statement

All animal experiments were conducted using protocols approved by the Animal Welfare and Ethical Review Boards of the Liverpool School of Tropical Medicine and the University of Liverpool. They were performed in specific pathogen-free conditions under licensed approval (PPL #4003718 and #P5846F90) of the UK Home Office and in accordance with the Animal (Scientific Procedures) Act 1986 and institutional guidance on animal care, which incorporate humane end point refinements to reduce the extent and duration of pain, harm and distress of the lowest possible number of experimental mice.

### Antivenom generation

#### Venom immunogens

Twelve venoms were sourced from a wide geographical and taxonomic diversity of snake species known to cause potent haemotoxicity in snakebite victims (Table 1). Many of these venoms were collected from animals maintained under controlled environmental and dietary conditions in the UK Home Office licensed and inspected herpetarium of the Centre for Snakebite Research and Interventions (CSRI) at the Liverpool School of Tropical Medicine. The remainder were sourced from historical venom collections held in the CSRI herpetarium or commercially purchased from Latoxan, France. Lyophilised venoms were stored at 4°C and reconstituted with phosphate-buffered saline (PBS) (0.12 M NaCl, 0.04 M phosphate, pH 7.2) to a concentration of 1 mg/ml prior to use. The same batches of these venoms were used for each of the analyses to provide cross-experimental continuity.

**Table 1:** The venoms used in the immunisation mixtures for the generation of experimental antivenoms (EAVs).

	Species	Sub-family	Geographical region	Venom origin
**Immunogen mixture I (resulting in EAV 1)**	*Bothrops asper*	Crotalinae	Central America	Costa Rica
	*Bothrops jararaca*	Crotalinae	South America	Brazil
	*Echis ocellatus*	Viperinae	West Africa	Nigeria
	*Calloselasma rhodostoma*	Crotalinae	Southeast Asia	Captive bred
	*Dispholidus typus*	Colubrinae	sub-Saharan Africa	South Africa*
	*Deinagkistrodon acutus*	Crotalinae	East Asia	Captive bred
	*Daboia russelii*	Viperinae	South Asia	Sri Lanka
**Immunogen mixture II (resulting in EAV 2)**	The same 7 venoms in immunogen mixture I plus:			
	*Bitis arietans*	Viperinae	sub-Saharan Africa	Nigeria
	*Echis carinatus*	Viperinae	Middle East & South Asia	India**
	*Rhabdophis subminiatus*	Natricinae	Southeast Asia	Hong Kong
	*Trimeresurus albolabris*	Crotalinae	Southeast Asia	Captive bred
	*Crotalus atrox*	Crotalinae	North America	USA

* venom sourced commercially from Latoxan, France

** Indian *Echis carinatus sochureki* venom (referred to as *Echis carinatus* throughout) was collected from a single specimen that was inadvertently imported to the UK via a boat shipment of stone, and then rehoused at LSTM on the request of the UK Royal Society for the Prevention of Cruelty to Animals (RSPCA).

#### Ovine immunisation

Two different venom mixtures were prepared as immunogens for antivenom generation. For each immunogen mixture, 3 mg of each venom was weighed and mixed manually in glass bijous, with immunogen mixture I consisting of seven procoagulant venoms from key medically important and/or geographically and taxonomically diverse snake species and mixture II consisting of the same seven venoms plus five additional haemotoxic venoms from snakes of secondary experimental importance (Table 1). The resulting mixtures were then reconstituted with PBS to a concentration of 1 mg/ml for immunisation. Ovine immunisation was performed by Ig-Innovations (Wales, UK) under licensed approval of the UK Home Office and in accordance with the UK Animal [Scientific Procedures] Act 1986, with n = 1 per immunogen group. Each sheep was initially immunised with equal quantities of an emulsion comprised of the venom mixtures (0.5 mg) and Freund’s Complete Adjuvant. Subsequent immunisations consisted of an emulsion of the venom mixtures (1.0 mg) and Freund’s Incomplete Adjuvant (1:1, volume/volume). Immunisations were performed every four weeks until 42 weeks. Low volume blood samples (~5 ml) were collected two weeks after each immunisation to monitor seroconversion over the immunisation time course, and at the end of the immunisation schedule (i.e. 42 weeks), a larger blood volume (~300 ml) was collected, and sera collected post-centrifugation for downstream IgG purification. Sera were stored long term at -20°C.

#### Immunoglobulin purification

For the purification of IgG from ovine serum [21], each of the 42-week serum samples (and a pre-immunisation sample to serve as a normal ovine IgG control) were first diluted with 0.90% saline (1:1 ratio), before the slow (drop by drop) addition of caprylic acid (Octanoic acid; Sigma, UK) with stirring to a final concentration of 6%. Subsequently, non-immunoglobulin proteins were precipitated by vigorous stirring for 60 minutes, followed by centrifugation at 12,800 x g for 60 min at 4°C. The resulting supernatant from each sample was dialysed separately using 35 mm 3.5K MWCO SnakeSkin Dialysis Tubing (Thermo-Fisher Scientific, UK) and sodium citrate buffered saline (SCS, pH 6.0), with two changes of SCS, first at room temperature (RT) for one hour, and then secondarily at 4°C overnight. Following dialysis, the final IgG samples were lyophilised using a Labogene lyophiliser and stored as a powder at 4°C prior to reconstitution in PBS. The resulting experimental antivenoms (EAV) were thereafter denoted as EAV 1 (generated from immunogen mixture I) and EAV 2 (generated from immunogen mixture II). For downstream testing, these EAVs were studied in comparison with the positive control antivenom used, the equine-derived SAIMR polyvalent antivenom (South African Vaccine Producers (PTY) Ltd, Gauteng, South Africa, expiry date: 11/2017, Lot#: BF00546, concentration 107 mg/ml), raised via immunisation with venom from *Bitis arietans*, *Bitis gabonica*, *Hemachatus haemachatus*, *Dendroaspis angusticeps*, *Dendroaspis jamesoni*, *Dendroaspis polylepis*, *Naja nivea*, *Naja melanoleuca*, *Naja annulifera* and *Naja mossambica*.

### *In vitro* immunological assays

#### SDS-PAGE gel electrophoresis and immunoblotting

For gel electrophoresis, 15 well 15% SDS-PAGE gels were prepared using the following approach: resolving gel (3.75 ml H_2_O, 2.5 ml Tris pH 8.8 [1.5 M], 3.75 ml 40% bis-acrylamide, 100 μl 10% SDS, 60 μl 10% ammonium persulfate [APS] and 7 μl N,N,N′,N′-Tetramethyl ethylenediamine [TEMED]); stacking gel (2.5 ml H_2_O, 1 ml Tris pH 6.8 [1 M], 350 μl 40% bis-acrylamide, 30 μl 10% APS and 5 μl TEMED). Next, 10 μl of each venom (1 mg/ml) was mixed 1:1 volume/volume with reducing buffer (150 μl β-mercaptoethanol and 850 μl of the following buffer mixture: 1.25 ml Tris pH 6.8 [0.5 M], 2.50 ml glycerol, 2.0 ml 10% SDS, 1.50 ml saturated Bromophenol blue) and subsequently heated for 10–15 minutes at 100°C. Thereafter, 10 μl of each venom/buffer sample was added to the gel, alongside 5 μl of a molecular weight protein marker (Broad Range Molecular Marker, Promega), and the samples run at 200 V for 55 minutes using a Mini-PROTEAN Electrophoresis System (Bio-Rad). Resulting gels were then stained at a final concentration of 0.1% (w/v) Coomassie blue R350 (0.4 g of Coomassie blue R350 in 200 mL of 40% [v/v] methanol in H_2_O, 10% (v/v) glacial acetic acid and 20% (v/v) methanol) overnight at RT. Gels were destained (4:1:5 methanol:glacial acetic acid:H_2_O) for at least 2 hours at RT. For visualisation, gels were subsequently imaged using a Gel Doc EZ Gel Documentation System (Bio Rad).

For immunoblotting, SDS-PAGE gel electrophoresis was performed as described above, but instead of Coomassie staining, proteins in the gels were transferred onto 0.2 μm nitrocellulose membranes using a Trans-Blot Turbo Transfer System (Bio-Rad). Following confirmation of protein transfer by reversible Ponceau S staining, the membranes were blocked for non-specific binding using 5% non-fat dried milk in TBST (0.15 M NaCl; 0.01 M Tris-HCl, pH 8.5; 1% Tween 20), and left overnight at 4°C rocking at slow speed. Subsequently, blots were washed three times over 15 minutes with TBST, before the addition of primary antibodies (either: EAV 1 or EAV 2, the negative control [pre-immunisation, naïve ovine IgG] or the positive control [SAIMR polyvalent antivenom]), with all primary antibodies standardised to an initial concentration of 50 mg/ml, and then diluted to 1:5,000 in 5% non-fat dried milk in TBST, and incubated for 2 hours at RT. The immunoblots were then washed in triplicate with TBST as described above and incubated for two hours at RT with 50 ml of appropriate secondary antibodies diluted 1:2,000 in PBS: (i) horseradish peroxidase-conjugated donkey anti-sheep IgG (Sigma, UK) for the two EAVs and the ovine IgG control and (ii) horseradish peroxidase-conjugated Rabbit anti-horse IgG (Sigma, UK) for the SAIMR Polyvalent positive control antivenom and the equine IgG control. The immunoblots were then washed again with TBST and developed by the addition of DAB substrate (50 mg 3,3’- 222 diaminobenzidine, 100 ml PBS and 0.024% hydrogen peroxide; Sigma) by placing the membrane into the substrate for 30 seconds, before washing with deionised water.

#### End-point titration by enzyme-linked immunosorbent assay (ELISA)

Microtiter 96 well ELISA plates (Thermo Fisher Scientific) were coated with coating buffer (100 mM carbonate/Bicarbonate buffer, pH 9.6) containing 100 ng of each venom or each venom immunogen mixture and incubated overnight at 4°C. The plates were then washed three times with TBST before the addition of 5% non-fat milk in TBST and incubated at RT for two hours, followed by washing another three times with TBST. Next, 120 μl of primary antibodies (as described for immunoblotting experiments) were added to the plate in duplicate at an initial dilution of 1:100 in 5% non-fat milk in TBST, followed by five-fold serial dilutions across the plate and incubation at 4°C overnight. The plates were then washed again with TBST and incubated for two hours at RT with appropriate secondary antibodies (as per immunoblotting experiments) diluted at 1:1,000 in PBS. The plates were then washed again with TBST before the addition of substrate (0.2% 2,2/-azino-bis[3-ethylbenzthiazoline-6-sulphonic acid]) in citrate buffer (0.5 M, pH 4.0) containing 0.015% hydrogen peroxide (Sigma, UK). Plates were gently mixed and incubated at RT for 15 mins, before the signal was read spectrophotometrically at 405 nm on a FLUOstar Omega microplate reader (BMG LabTech).

#### Relative avidity by ELISA

Relative avidity ELISAs were performed as per the end point titration ELISA assays, except that: (i) the primary antibodies were incubated at a single defined dilution (1:10,000) and (ii) after washing the primary antibody with TBST, wells were exposed to the chaotropic agent, ammonium thiocyanate (NH_4_SCN), at a range of concentrations (0–8 M) in duplicate for 15 minutes at RT [24]. The plates were then washed with TBST, and all subsequent steps were the same as described for the end point titration ELISA. To permit direct and informative comparisons, reduction percentages (in terms of OD values) were calculated by subtracting 4 M NH_4_SCN readings from 0 M readings (the control) and multiplying by 100.

### *In vitro* functional assays

#### Serine protease assay

To quantify venom serine protease activity and inhibition by the EAVs, we used a chromogenic kinetic enzymatic assay [43]. One microgram (1 mg/ml) of each venom and 10.6 μg (70 mg/ml) of the two EAVs or the commercial antivenom (SAIMR polyvalent) were co-incubated in a water bath at 37°C for 30 min. This incubation time (and as used for other assays below) was selected to match the venom-antivenom incubation times conventionally applied across preclinical antivenom efficacy studies (see later). For determining baseline venom activity, we replaced antivenom with PBS, to act as a venom only control. A positive control venom with known serine protease activity was also used for standardisation across assays (1 μg; *B*. *arietans* [43]), alongside a PBS (no venom) negative control. Each sample was added in triplicate to a 384-well microplate (Greiner). The plate was then incubated for 3 min at 37°C in a FLUOstar Omega microplate reader (BMG Labtech). Next, 15 μl of buffer (100 mM Tris, pH 8.5, 100 mM NaCl) was overlaid using a pipetting robot (Multidrop, Thermo Scientific) and the plate incubated for a further 3 min at 37°C. Finally, 15 μl of 6 mM chromogenic substrate (S-2288, Cambridge Bioscience) at a final concentration of 2 mM was added. The enzymatic reaction was then monitored kinetically for 60 cycles (30 sec. each; ~30 min total) using a FLUOstar Omega microplate reader (BMG Labtech), with a temperature setting of 37°C and using a wavelength of 405 nm. Mean measures of absorbance were plotted against time to compare venom activity with baseline (negative controls) and positive control readings. For quantification, we first calculated the mean rates (expressed as ΔAbs/time/μg venom) of at least triplicate readings by subtracting negative control readings (PBS) from each reading. The rate of substrate consumption was calculated by subtracting the 0 min readings from the 5 min readings and then dividing by 5 min. Finally, the reduction percentage for all antivenom samples was calculated by subtracting the mean of the relevant negative control readings from the venom readings. The resulting triplicate readings were then plotted with standard error of the means (SEMs).

#### Metalloproteinase assay

To quantify snake venom metalloproteinase activity and inhibition by the EAVs, we used a fluorescent kinetic enzymatic assay [44]. The SVMP assay kinetically measured cleavage of a quenched fluorogenic substrate (ES010, R&D Biosystems) by venom in the presence or absence of inhibitors. One microgram (1 mg/ml) of each venom and 6.91 μg (70 mg/ml) of the two EAVs or the commercial antivenom (SAIMR polyvalent) were co-incubated in a water bath at 37°C for 30 min. For determining baseline venom activity, we replaced antivenom with PBS, to act as venom only controls. A positive control venom with known metalloproteinase activity was also used for standardisation across assays (1 μg; *E*. *ocellatus* [44]), alongside a PBS (no venom) negative control. Ten microlitres of each sample was then added in triplicate to a 384-well microplate (Greiner) followed by the addition of 90 μl of the substrate (10 μl of the substrate [supplied as a 6.2 mM stock] was used per 5 ml reaction buffer [50 mM Tris-Cl pH 7.5, 150 mM NaCl] at a final substrate concentration of 10 μM), to all samples using a multichannel pipette. The assay was then read kinetically for 1 hour at 25°C using a FLUOstar Omega microplate reader (BMG Labtech) and an excitation wavelength of 320 nm and an emission wavelength of 405 nm. The enzymatic reaction was monitored by setting the gain adjustment on the instrument to 5% in wells where high activity was expected (e.g., positive control/venom only mixtures). Mean measures of absorbance were then plotted against time to compare venom activity with baseline (negative controls) and positive control readings. For quantification, we calculated areas under the curves (AUCs) and the standard error of mean AUCs readings for each sample in the 0–40 min interval, this time point was chosen as the time where all fluorescence curves had reached a plateau (maximum fluorescence). We then subtracted the mean of the relevant negative control readings from the venom readings and calculated the reduction percentage for all antivenom samples and plotted the triplicate readings with SEMs.

#### Plasma coagulation assay

To quantify venom coagulotoxicity and inhibition by the EAVs, we used a kinetic absorbance-based coagulation assay [45]. One microgram (100 ng/μl) of each venom and 3.75 μg (70 mg/ml) of the two EAVs or the commercial positive control antivenom (SAIMR polyvalent) were co-incubated in a water bath at 37°C for 30 min. The positive control used was 1 μg of *E*. *ocellatus* venom based on its potent procoagulant effect in this assay [44], and the negative control was plasma with the addition of PBS instead of venom. During sample incubation, bovine plasma (sterile filtered, Biowest, Nuaille, France) was defrosted and centrifuged for 4 min at 805 x g to remove precipitate prior to use. Following incubation, 10 μl of each venom-antivenom mixture was added to a 384 well microplate (Greiner), followed by the addition of 20 μl of 20 mM CaCl_2_ (prepared fresh for each assay), and finally 20 μl of the citrated bovine plasma. The plates were loaded using a multidrop pipetting robot (Multidrop Labsystems) and resulting clotting data were captured kinetically for 2 hours using a FLUOstar Omega microplate reader (BMG Labtech) at OD 595 nm and 25°C. Mean measures of absorbance were plotted against time to compare venom activity with baseline (negative controls) and positive control readings. For quantification, we calculated the AUCs and the standard error of the mean AUCs. We then subtracted the mean of the relevant negative control readings from the venom readings and calculated the reduction percentage of venom activity for all datasets with SEMs.

### *In vivo* efficacy assays

#### *In vivo* venom median lethal dose

To assess the preclinical efficacy of the EAVs, we used modified versions of established murine models of envenoming [44] and selected a sub-set of the venoms used as immunogens, along with two additional venoms not used as immunogens, to assess the breadth of antivenom paraspecific efficacy against haemotoxic snakebite. As an essential prerequisite to preclinically assessing antivenom efficacy, World Health Organization-recommended protocols were used to determine the median murine lethal dose (LD_50_) of various snake venoms via the intravenous route [44]. For these studies, we selected four venoms used as immunogens for generating both EAVs (*Bothrops asper*, *Calloselasma rhodostoma*, *E*. *ocellatus* and *Daboia russelii*) and two venoms used as immunogens only for generating EAV 2 (*B*. *arietans* and *Echis carinatus*). We also used venom from two species of snake not used as venom immunogens for either EAVs to assess their paraspecific neutralisation capabilities, specifically *Vipera ammodytes* (captive bred) and *Lachesis muta* (Brazil). Existing LD_50_ data was available for six of the eight venoms used (Table 2) and, for each of these six venoms, the described LD_50_ data had previously been applied to the same venom samples as those used in this study in other studies previously conducted by the authors [43,44,46] Therefore, LD_50_ experiments were only performed for venom samples from *C*. *rhodostoma* and *L*. *muta*, as these had not previously been utilised for *in vivo* research. All experimental animals (4–5 weeks old, 18–20 g, male, CD-1 mice, Charles River, UK) were housed in groups of five with environmental enrichment, water and food *ad libitum* and their health monitored daily during acclimatisation. Each animal received an iv tail injection of varying doses of venom in 100 μl PBS, and experimental animals were monitored for 6 hours after injection, with the number of surviving mice in each group recorded. Experimental animals were euthanised on ethical grounds via rising concentrations of CO_2_ if they exhibited signs of humane end points that are predicators of lethality (e.g., pulmonary distress, immobility, seizure, haemorrhage, paralysis) [44]. The resulting LD_50_ values and 95% confidence intervals were generated based on dose and mortality data using Probit analysis [47].

**Table 2:** The murine intravenous median lethal dose (LD_50_) of the haemotoxic snake venoms used in this study.

Species	Venom origin	LD_50_ (μg/mouse)	95% confidence intervals (μg)	Reference
*Bothrops asper*	“Atlantic”, Costa Rica	18.8	(15.6–22.0)	[61]
*Calloselasma rhodostoma*	Thailand	31.2	(20.6–47.2)	This study
*Echis ocellatus*	Nigeria	20.1	(17.3–22.8)	[62]
*Daboia russelii*	Sri Lanka	7.8	(7.0–8.6)	[63]
*Bitis arietans*	Nigeria	21.6	(20.0–29.0)	[48]
*Echis carinatus*	India	19.0	(16.0–24.0)	[48]
*Vipera ammodytes*	Captive bred	8.0	(7.5–8.5)	[46]
*Lachesis muta*	*Brazil*	6.8	(5.4–8.5)	This study

#### *In vivo* antivenom efficacy

To assess the efficacy of the EAVs at protecting experimental animals from the lethal effects of envenoming, we used a modified version of the median effective dose (ED_50_) assay [48]. For each of the eight venoms, the procedure consisted of groups of five mice (4–5 weeks old, 18–20 g, male, CD-1 mice, Charles River, UK; maintained as described above) receiving an iv tail injection of either: (i) 5 x LD_50_ dose of venom only, or 5 x LD_50_ dose of venom preincubated for 30 mins at 37°C with 100 μl (5 mg) of (ii) EAV 1, (iii) EAV 2 or (iv) SAIMR polyvalent (as a positive control) antivenom. Antivenom concentrations were standardised to 50 mg/ml prior to use, and all final injected doses were made up to 200 μl using PBS (0.12 M NaCl, 0.04 M phosphate, pH 7.2). As detailed above, experimental animals were monitored for signs of suffering for 6 hours, with euthanasia applied via the implementation of the described humane endpoints where necessary, and the number of deaths, time of euthanasia and survivors in each group recorded throughout the experiment. This monitoring window was selected to capture protection against acute venom toxicity, and applied based on animal ethical grounds to reduce the duration of severe suffering imposed on experimental animals during envenoming experiments. Thereafter, Kaplan–Meier survival graphs were used to visualise the relative preclinical protection provided by the antivenoms against each of the venoms.

### Statistical analyses

To statistically test whether the EAVs inhibited the *in vitro* activity of each venom in the serine protease, metalloproteinase and plasma clotting assays we compared mean readings with venom only control readings using one-way ANOVA (with Dunnett’s multiple comparisons test) and a significance threshold of (*P*≤0.05). All statistical analyses were performed using Prism v8 software (GraphPad).

## Results

### Monitoring seroconversion during immunisation

**Fig 1:**
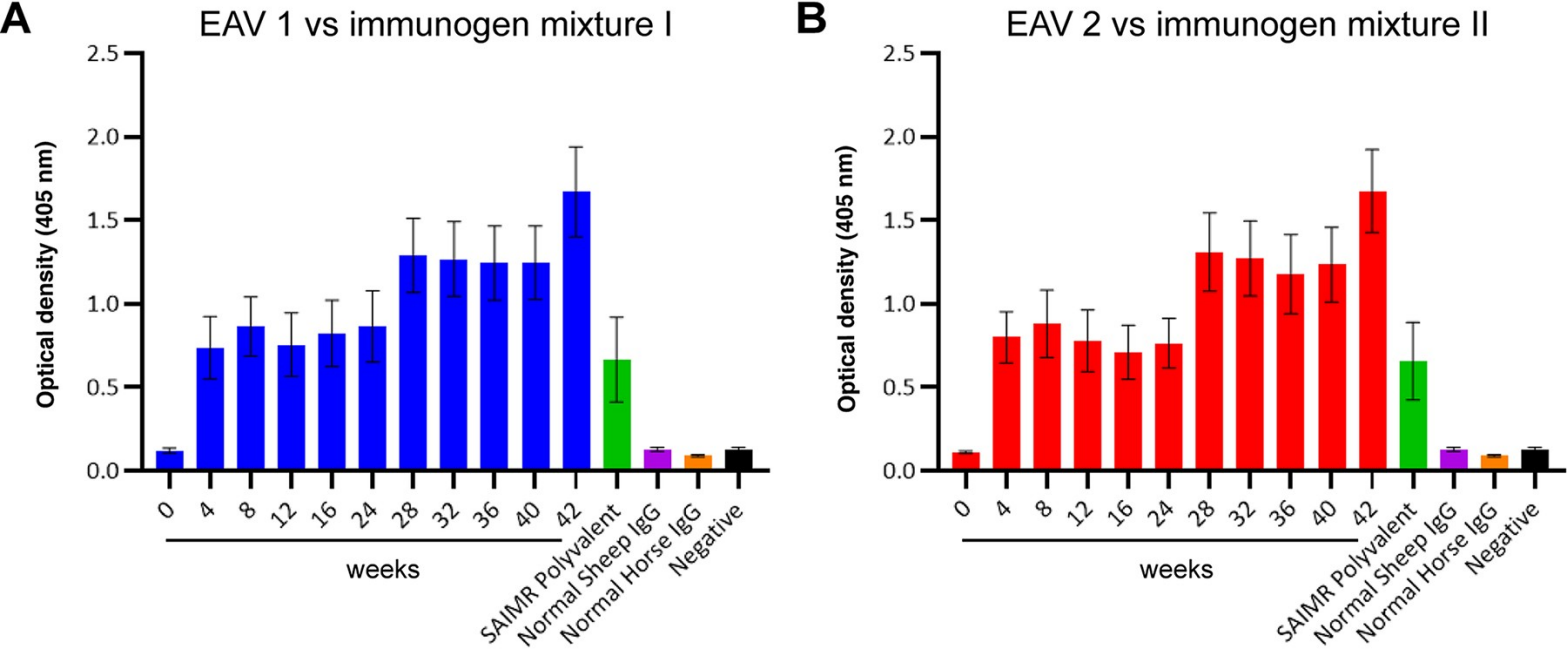
Time course analysis of the immunological cross-reactivity of ovine serum to the immunogen mixtures over 42 weeks of immunisation. **A)** Responses of EAV 1 against immunogen mixture I (containing seven venoms) and **B)** responses of EAV 2 against immunogen mixture II (containing 12 venoms). All sheep serum samples were standardised to 50 mg/ml prior to a 1:1,000 dilution for use and compared with a variety of appropriate controls: normal IgG sourced from non-immunised sheep and horses, the commercial equine antivenom SAIMR polyvalent and a PBS (no venom) negative control. Data points represent means of duplicate readings and error bars represent standard deviation (SD) of the duplicate measurements.

To assess the specificity of the immune response towards the venom immunogens over the course of the immunisation period, serum samples were collected every four weeks and assessed via ELISA to quantify antibody binding levels. Both sheep responded in a highly similar manner to the different immunogen mixtures (see Table 1 for details of immunogens), with rapid increases in antibody binding levels observed following primary immunisation, followed by a short plateau in antibody titres and then secondary increases (Fig 1). For both immunised animals, serum samples collected at the end of the immunisation schedule (week 42) exhibited the highest antibody binding titres (Fig 1). These samples were subsequently subjected to IgG extraction for preparation of EAV 1 and EAV 2.

### Immunological cross-reactivity of the experimental antivenoms

**Fig 2:**
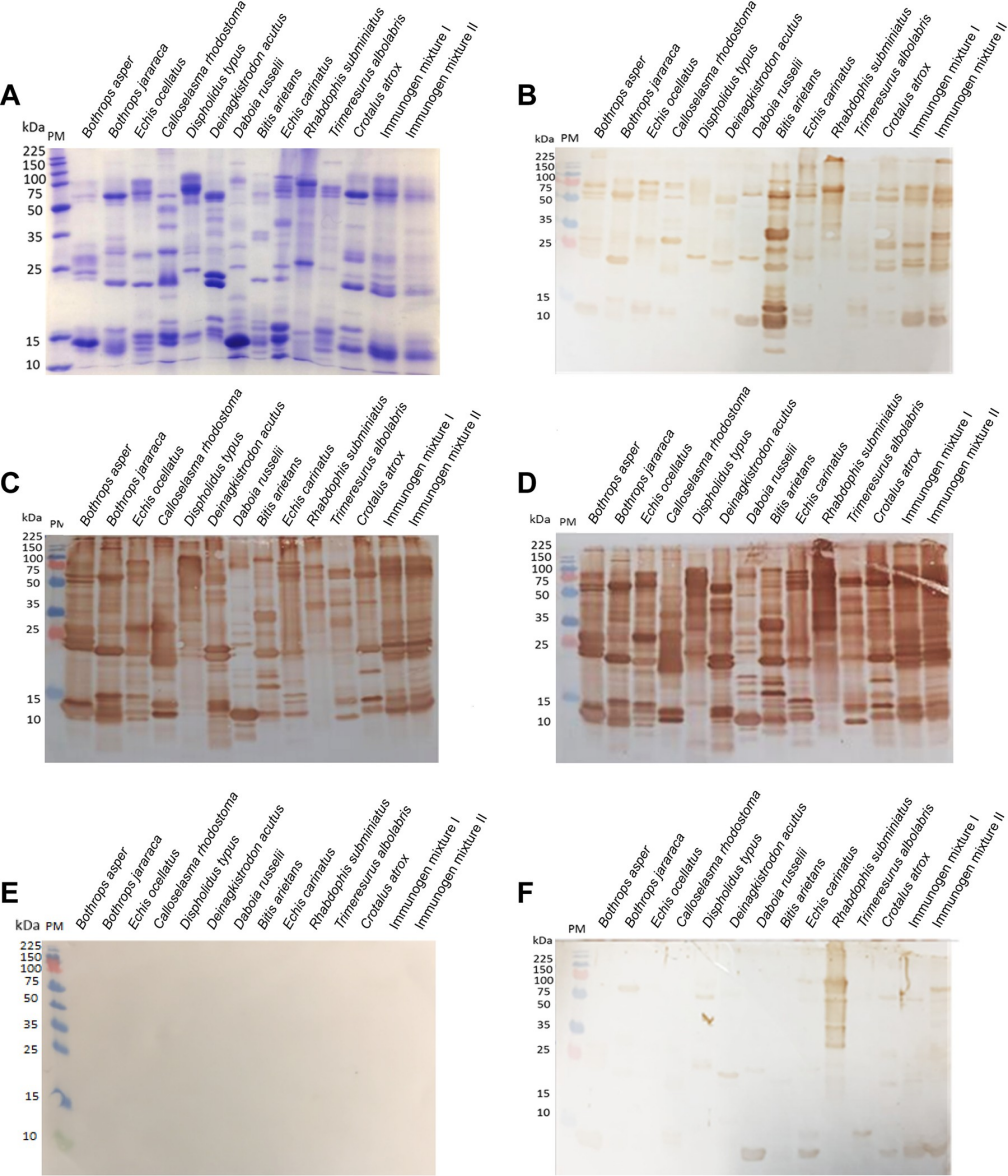
SDS-PAGE gel electrophoretic profiles of the individual venoms and the venom immunogen mixtures and their immunological recognition by experimental antivenoms. **A)** Venoms were separated by reduced SDS-PAGE gel electrophoresis and visualised by Coomassie blue staining. The same venom samples were transferred to nitrocellulose membranes for immunoblotting, and incubated with 1:5,000 dilutions of SAIMR polyvalent antivenom (positive control) (**B**), EAV 1 (**C**), EAV 2 (**D**), normal horse IgG (**E**) and normal sheep IgG (**F**) (both negative controls). PM indicates protein marker.

To visualise the immunological recognition of the two EAVs against the venom proteins found in the 12 venoms used in the immunisation process, we subjected each venom and each venom immunogen mixture to reduced SDS-PAGE gel electrophoresis and western blotting. As anticipated, the reduced SDS-PAGE profiles illustrated considerable inter-specific variation in the molecular weights and relative abundances of the different proteins found in the various venoms (Fig 2A). Immunoblotting experiments with each of the EAVs revealed extensive immunological recognition of the proteins found in each of the snake venoms and the two immunising mixtures, despite EAV 1 being generated with only seven of the 12 venoms used as immunogens to generate EAV 2 (Fig 2C and 2D). However, despite these broad similarities, these experiments revealed that EAV 1 exhibited some reductions in binding intensity compared with EAV 2, and also did not recognise the low molecular weight components (i.e., 10–15 kDa) of *D*. *typus* and *R*. *subminiatus* venoms to the same extent as EAV 2. As anticipated, both EAVs exhibited increases in venom recognition compared with the SAIMR polyvalent antivenom (positive control, and for which only *B*. *arietans* is an immunogen; Fig 2B) and the normal horse and sheep negative controls (Fig 2E and 2F).

**Fig 3:**
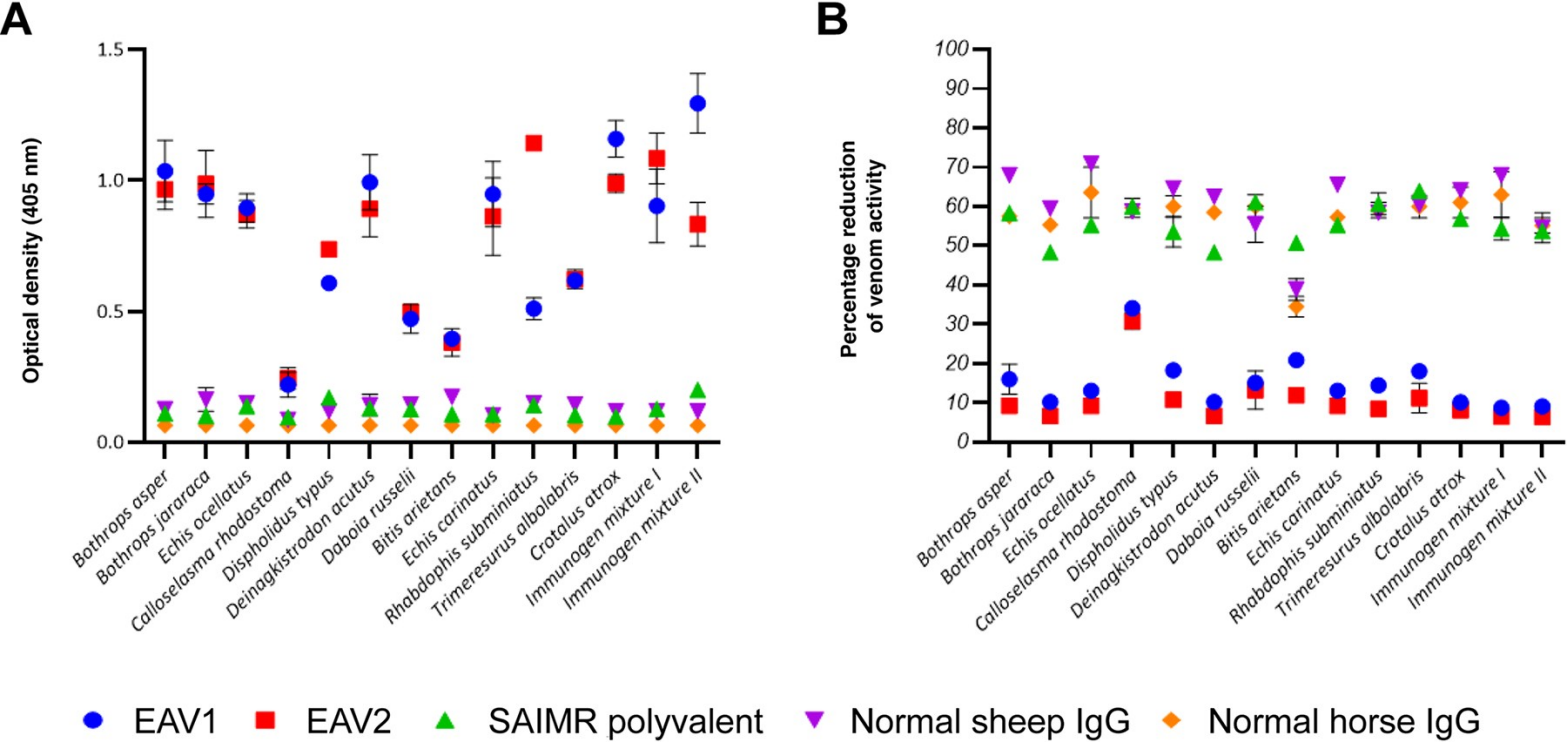
A comparative summary of the immunological binding observed between the two experimental antivenoms and the individual venoms and venom immunogen mixtures as measured by end-point and avidity ELISA. **A)** A summary of the immunological binding observed by end-point titration ELISA. The data displayed represents the mean optical density following the addition of primary antibodies at 1:1000 dilution. See S1 and S2A Figs for full binding profiles generated using fivefold titrations. **B)** A summary of the immunological binding observed by avidity ELISA. The data displayed represents the mean percentage reduction in antibody binding levels for each venom measured in the presence of 4M and 0M (control) ammonium thiocyanate. See S2B and S3 Figs for full binding profiles at increasing concentrations of the chaotrope (0-8M). For both sets of experiments, the primary antibodies consisted of EAV 1 and EAV 2, the commercial SAIMR polyvalent antivenom positive control, and normal sheep and horse IgG as negative controls. Error bars represent SD of duplicate measurements.

Next, we used end-point titration ELISAs to quantify the binding between the EAVs and the various venom immunogens. Comparisons of the binding titres of each EAV revealed highly comparable levels to each of the individual venoms used as immunogens (Figs 3A and S1), despite five additional venoms being added to the immunogen mixture used to raise EAV 2. An exception to this was that EAV 2 exhibited considerably highly titres to the venom of *R*. *subminiatus* than EAV 1 (Figs 3A and S1). This finding is consistent with the presence of this venom only being in the immunising mixture used to raise EAV 2, though, interestingly, no such differences were observed with the other four venoms unique to this immunogen mixture (i.e. *B*. *arietans*, *E*. *carinatus*, *Trimeresurus albolabris* and *Crotalus atrox*). Secondly, and perhaps surprisingly, we observed substantial increases in immunological binding to immunogen mixture II with EAV 1, rather than EAV 2 (Figs 3A and S2A). When comparing the levels of immunological cross-reactivity from the venom perspective, we observed considerable variation in binding levels, with six venoms exhibiting high titres, five with more moderate levels of binding and perhaps the most noticeable finding being that only low levels of binding were observed between *C*. *rhodostoma* venom and both EAVs (Figs 3A and S1).

To determine the strength of these antibody-venom protein binding interactions we performed avidity ELISAs, which consisted of quantifying venom-antibody binding levels in the presence of increasing concentrations of a chaotropic agent (ammonium thiocyanate, NH_4_SCN; 0-8M) that disrupts antibody-antigen binding. The results revealed that both EAVs exhibit highly comparable, and potent, binding to the two venom mixtures and to each individual venom used as an immunogen, even in the presence of up to 4M ammonium thiocyanate (Figs 3B, S2B and S3). For the vast majority of venoms, the binding reductions observed at 4M concentrations of the chaotrope were less than 20% of those observed with the control (no chaotrope). The exception to this was the venom of *C*. *rhodostoma*, which, as also observed in the end-point ELISA experiments, exhibited lower binding levels with both EAV 1 and EAV 2, and resulted in percentage reductions of 35% and 30%, respectively (Figs 3B and S3). Comparisons between EAV 1 and EAV 2 revealed very little difference in avidities across the individual venoms and the venom immunogen mixtures, though in general EAV 2 exhibited slightly lower reductions in antibody binding in the presence of the chaotrope compared with EAV 1 (Figs 3B, S2B and S3).

### *In vitro* venom neutralisation by the EAVs

While high levels of immunological binding appear to be a prerequisite for antivenom efficacy, immunological binding does not necessarily result in toxin neutralisation. To assess the capability of the EAVs to neutralise venom functional activities related to haemotoxicity, we employed three different *in vitro* functional screening assays, which specifically related to SVSP, SVMP and coagulotoxic venom activities.

**Fig 4:**
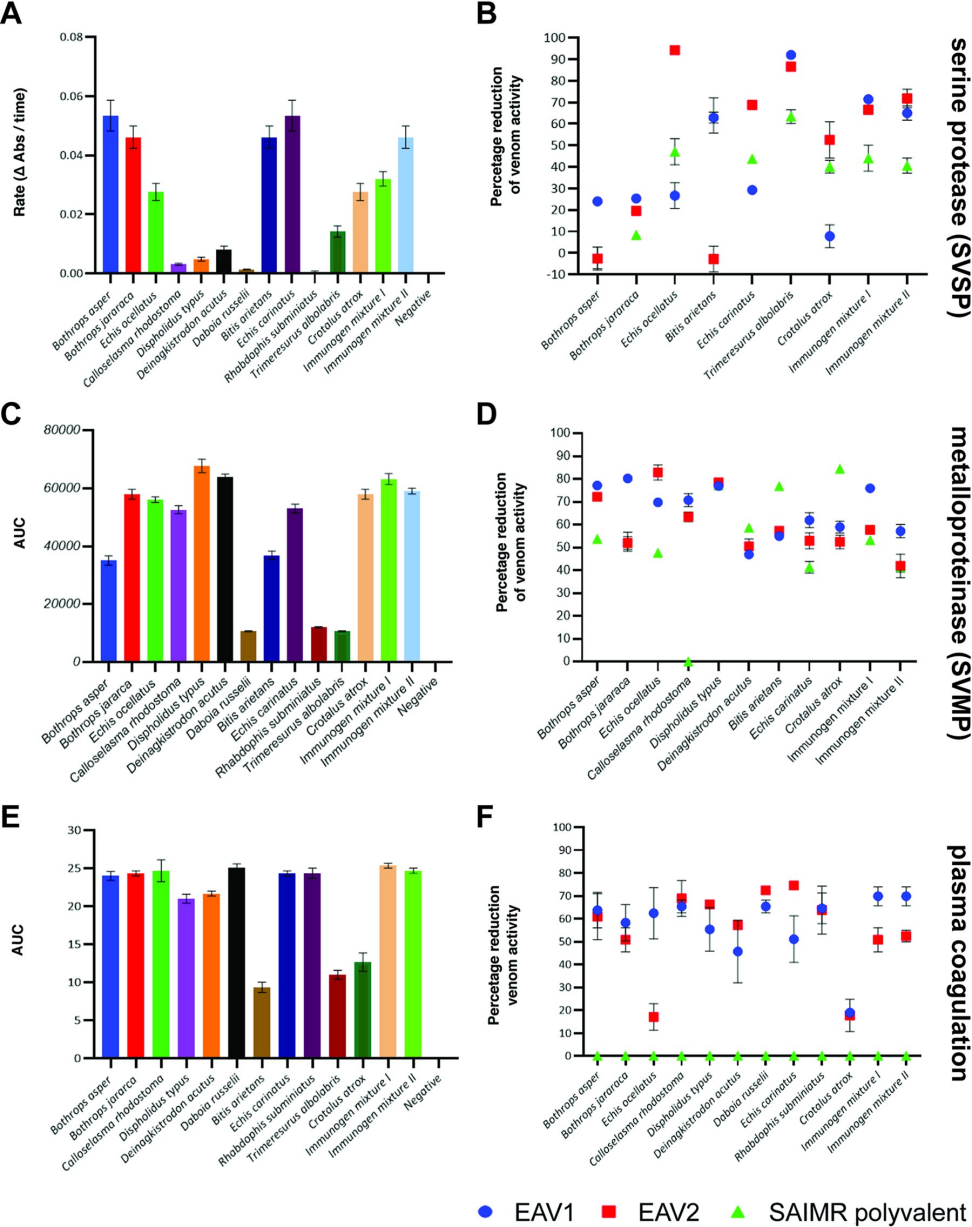
The *in vitro* venom activity of the venom immunogens and quantification of venom neutralisation by the experimental antivenoms. **A**) The serine protease (SVSP) activity of individual venoms and the venom immunogen mixtures displayed as the rate of cleavage (absorbance/time) of the chromogenic substrate. **B**) Inhibition of venom serine protease activities by the EAVs displayed as the percentage reduction of venom only activities from (**A**). Also see S2C and S4 Figs. **C)** The metalloproteinase (SVMP) activity of individual venoms and the venom immunogen mixtures displayed as the area under the curve (AUCs) resulting from cleavage of a fluorescent substrate over time. **D)** Inhibition of venom metalloproteinase activities by the EAVs displayed as the percentage reduction of venom only activities from (**C**). See also S2D and S5 Figs. **E)** The coagulopathic activity of individual venoms and the venom immunogen mixtures displayed as the AUCs resulting from increases in absorbance stimulated by clotting over time. **F)** Inhibition of coagulopathic venom activities by the EAVs displayed as the percentage reduction of venom only activities from (**E**). See also S2E and S6 Figs. For all data shown, data points represent means of triplicate readings, and error bars represent standard error of the mean (SEMs).

The results of our kinetic chromogenic SVSP assay revealed that seven of the 12 venoms tested, and both of the venom immunogen mixtures, exhibited considerable SVSP activity as evidenced by substantial increases in the rate of substrate cleavage compared to the negative control (Figs 4A and S4). These venoms included three used as venom immunogens for both EAVs (*B*. *asper*, *Bothrops jararaca*, *E*. *ocellatus*) and four used only to raise EAV 2 (*B*. *arietans*, *E*. *carinatus*, *T*. *albolabris* and *C*. *atrox*). Consequently, we noted that venom immunogen mixture II exhibited increased SVSP activity compared with mixture I (Figs 4A and S2C). The other venoms had little to no SVSP activity at the concentration tested, and thus were not used in subsequent neutralisation experiments using the EAVs. Neutralisation of SVSP activity at the tested antivenom concentration showed a variable pattern across the venoms, with EAV 2 exhibiting high percentage reductions (>50% of venom only activity) against four of the seven individual venoms tested, but had no effect on *B*. *asper* or *B*. *arietans* venom, and only resulted in minor reductions against *B*. *jararaca* (Figs 4B and S4). Contrastingly, EAV 1 only exhibited high levels of SVSP inhibition against *B*. *arietans* and *T*. *albolabris–*a surprising result since neither of these venoms were included in the immunogen mixture used to generate this EAV (Figs 4B and S4). More moderate reductions (20–30%) in SVSP activity were observed for four of the remaining venoms. Despite these observations, both EAVs exhibited highly comparable inhibition of the SVSP activity of the two immunogen mixtures, and though neither neutralised the venom activity to control readings, both exhibited significant inhibition (*P*≤0.05 for all comparisons vs venom only controls, one-way ANOVA), resulting in >60% reductions in SVSP activity (Figs 4B and S2C).

The enzymatic SVMP activities of the venoms used in this study were quantified via a kinetic, fluorescent assay. The majority of the 12 venom immunogens exhibited detectable SVMP activity, with the exceptions being those from *R*. *subminiatus*, *T*. *albolabris* and *D*. *russelii* (Figs 4C and S5). The latter finding is perhaps surprising given that a potent Factor X activating SVMP is known to be present in *D*. *russelli* venom [13], suggesting that this result may perhaps be a reflection of limitations in substrate specificity. Nonetheless, SVMP inhibition studies against the remaining nine venoms revealed that both EAVs exhibited considerable neutralising capability, with between 40–80% of the venom SVMP activity reduced across the various venoms (Figs 4D and S5). Detailed comparisons between both EAVs revealed highly similar percentage reductions in SVMP activity for six of the nine venoms tested (*B*. *asper*, *D*. *typus*, *Deinagkistrodon acutus*, *B*. *arietans*, *E*. *carinatus* and *C*. *atrox*), while EAV 1 exhibited modest increases in inhibitory potency against *B*. *jararaca* (80.2% reduction in venom only activity), though EAV 2 slightly outperformed EAV 1 against *E*. *ocellatus* venom (83.3% vs 70.1% reduction, respectively) (Figs 4D and S5). Interestingly, the SAIMR polyvalent control antivenom outperformed both EAVs in inhibiting *B*. *arietans* and *C*. *atrox* venoms, though the former of these two venoms is used as an immunogen during the generation of this product. This control antivenom also exhibited comparable SVMP neutralising capabilities to the EAVs against *D*. *acutus* and *D*. *typus* venoms, though it was much less effective against *B*. *asper* and *E*. *ocellatus* (Figs 4D and S5). Against the two immunogen mixtures, all three antivenoms tested exhibited significant reductions in venom activity compared with control readings (*P*≤0.05 for all comparisons vs venom only controls, one-way ANOVA). While there was little difference between the levels of inhibition observed between EAV 2 and the SAIMR polyvalent control antivenom, EAV 1 outperformed both comparators, resulting in percentage reductions of 75.9% and 57.1% of SVMP activity for immunogen mixture I and II, respectively (vs 57.7% and 41.5% for EAV 2) (Figs 4D and S2D).

To assess the coagulopathic activity of the venom immunogen mixtures and their constitutive venoms, we used an absorbance-based plasma clotting assay. Nine of the 12 venom immunogens and both venom mixtures exhibited potently procoagulant activities with an additional venom (*C*. *atrox*) exhibiting moderate procoagulant activity, as evidenced by increases in kinetic profiles compared with the negative control (Figs 4E and S6). Neither *B*. *arietans* nor *T*. *albolabris* exhibited potent coagulopathic activity in this assay at the concentration tested. Neutralisation experiments with the two EAVs revealed marked inhibition of the coagulopathic toxins found in the majority of the coagulopathic venom immunogens (Figs 4F and S6). With the exception of *C*. *atrox*, EAV 1 reduced the procoagulant activity of the other nine venoms tested by 50–70%, including a number of venoms not used as immunogens to raise this antivenom (e.g. *R*. *subminiatus* and *E*. *carinatus*) (Figs 4F and S6). The results observed with EAV 2 were highly comparable to those of EAV 1 (percentage reductions of 50–70% for eight of the ten tested venoms), although in addition to a comparable lack of inhibition against *C*. *atrox*, EAV 2 also lacked inhibitory potency against the venom of *E*. *ocellatus* (percentage reduction of 20%), despite this venom being included in the immunising mixture (Figs 4F and S6). Both venom immunogen mixtures were also found to be potently procoagulant, and this venom activity was effectively neutralised by both EAV 1 and EAV 2, although inhibition was greatest with EAV 1, irrespective of the venom immunogen mixture (75.9% reduction vs 57.7% against immunogen mixture I; 57.1% vs 41.5% against immunogen mixture II) (Figs 4F and S2E). However, despite neither EAV by itself affecting normal coagulation, the addition of EAV 1 in the presence of the two venom immunogen mixtures resulted in a delay of clotting, and therefore net anticoagulant activity (S2E Fig). The reason for this remains unclear, but might indicate potent inhibition of procoagulant venom toxins, but ineffective inhibition of anticoagulant venom toxins, as seen elsewhere [43]. Contrastingly, EAV 2 effectively inhibited venom immunogen stimulated coagulation to control levels, though this same phenomenon was observed for both EAVs against many of the individual venom immunogens (S6 Fig). The SAIMR polyvalent antivenom was ineffective against all of the individual venoms and venom immunogen mixtures tested, likely as the result of no procoagulant snake venoms being used as immunogens during the production of this product (Fig 4F).

### *In vivo* neutralisation of venom lethality

Following promising evidence of *in vitro* immunological cross-reactivity and venom neutralisation with the two EAVs, we next assessed their preclinical efficacy against a subset of haemotoxic venoms using a variation of the World Health Organization-recommended assay for assessing antivenom efficacy, the murine median effective dose [49]_._ We selected eight venoms for preclinical assessment; four that were included in both venom immunogen mixtures (*B*. *asper*, *C*. *rhodostoma*, *E*. *ocellatus* and *D*. *russelii*), two that were only included in the immunogen mixture used to generate EAV 2 (*B*. *arietans* and *E*. *carinatus*) and, to assess whether the breadth of antibodies generated by the diverse immunogen mixtures stimulated broad paraspecific efficacy, two haemotoxic venoms that were not used in either immunogen mixture (*V*. *ammodytes* and *L*. *muta*) (Table 2). Prior to performing antivenom efficacy experiments, the LD_50_ of *L*. *muta* and *C*. *rhodostoma* venoms were determined, resulting in LD_50_s of 6.8 μg/mouse (95% confidence intervals: 5.40–8.54 μg/mouse) and 31.2 μg/mouse (20.6–47.2 μg/mouse), respectively. The LD_50_s for the remaining species were sourced from previously published studies (Table 2). Thereafter, groups of five mice were dosed with 5 x LD_50_ dose of venom only, or 5 x LD_50_ dose of venom preincubated for 30 mins at 37°C with 100 μl (5 mg) of either EAV 1 or EAV 2, or the control SAIMR polyvalent antivenom (all were standardised to 50 mg/ml).

**Fig 5:**
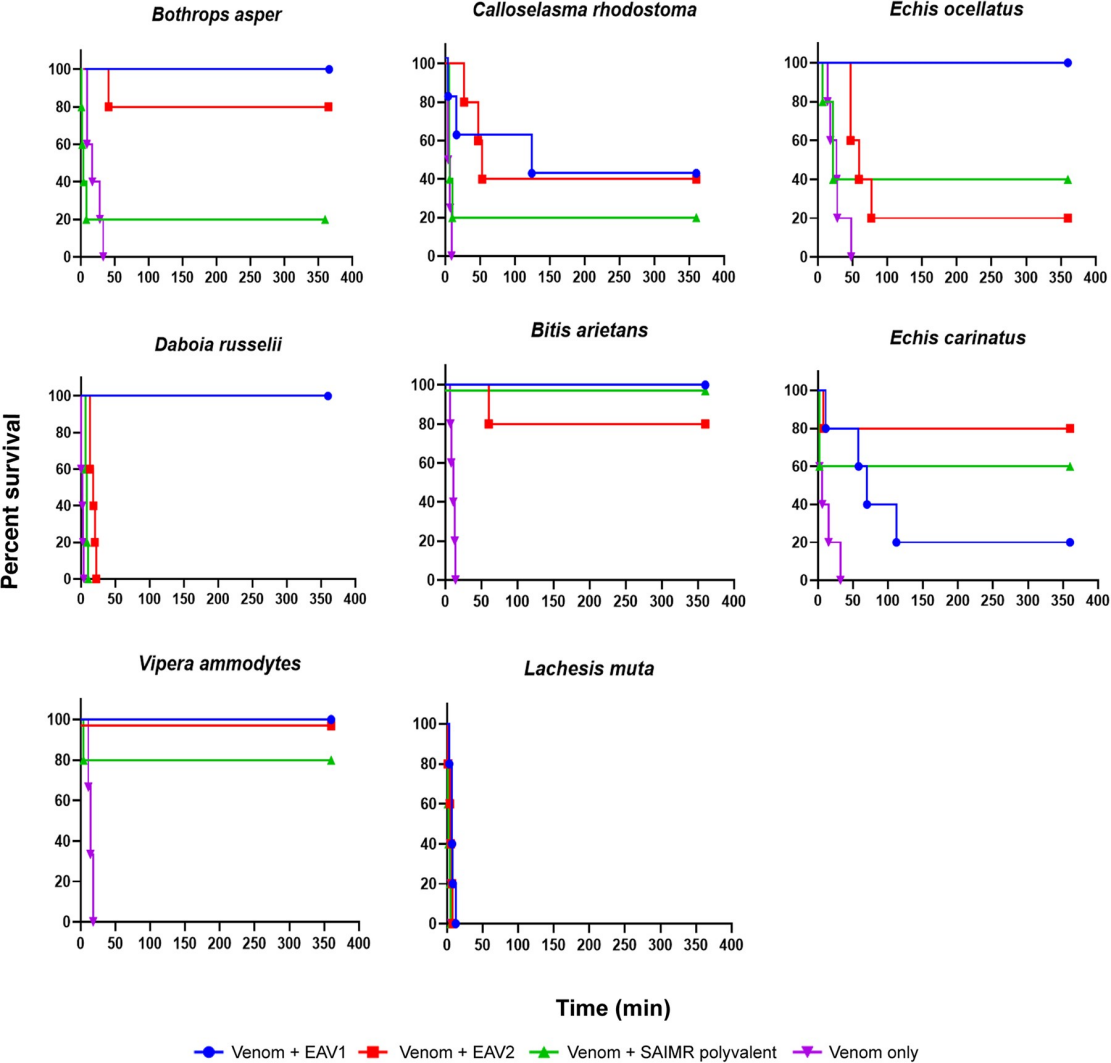
Kaplan–Meier survival curves for murine preclinical efficacy experiments using representative, geographically diverse, haemotoxic snake venoms and the experimental antivenoms. Groups of five mice were challenged intravenously with 5 x LD_50_ doses of each venom (purple lines) or venom preincubated for 30 min at 37°C with EAV 1 (blue) or EAV 2 (red), or the SAIMR polyvalent control antivenom (green). All experimental animals were monitored for 6 hours and survival times recorded. All antivenom doses were standardised to 5 mg (100 μl, 50 mg/ml).

Our dose-matched pilot *in vivo* findings demonstrated that, despite a reduction in the number of venoms used in the immunogen mixture (7 vs 12), EAV 1 outperformed EAV 2 in terms of preclinical efficacy (superior vs four venoms; equipotent vs two venoms; inferior vs one venom) (Fig 5). Indeed, EAV 1 effectively prevented venom-induced lethality for the duration of the experiment against three of the four venoms used as immunogens (*B*. *asper*, *E*. *ocellatus* and *D*. *russelii*), and two of the four venoms tested that were not present in the immunising mixture (*B*. *arietans* and *V*. *ammodytes*) in this model (Fig 5). Contrastingly, none of the venoms present in the immunogen mixture used to raise EAV 2 were fully neutralised (i.e. conferred complete protection, 100% survival) by this EAV, though four of the five experimental animals survived the challenge dose with *B*. *asper*, *B*. *arietans* and *E*. *carinatus* venom (Fig 5). The result with this latter venom proved to be the only example of superior preclinical efficacy exhibited by EAV 2 over EAV 1 (four vs one survivor, respectively). Disappointingly, EAV 2 exhibited limited/no protection against envenoming by *E*. *ocellatus* and *D*. *russelii–*arguably the two most medically-important species tested (Fig 5). Both EAVs exhibited comparable, limited protection against the lethal effects of *C*. *rhodostoma* venom (two of five experimental animals survived the duration of the experiment), which correlates with our earlier observations of reduced immunological cross-reactivity to the toxins found in this venom (see Fig 3), and suggests that higher therapeutic doses than tested here are likely required to provide full protection. Identical and contrasting efficacy observations were also observed in respect of the remaining two venoms tested, neither of which was used as an immunogen to generate either EAV; both antivenoms failed to provide any protection against the lethal effects of *L*. *muta* venom, though both EAVs exhibited potent paraspecific preclinical efficacy against *V*. *ammodytes* (Fig 5). To place these findings into context, we compared our preclinical efficacy data against that obtained using our antivenom control, SAIMR polyvalent, which is manufactured using venom from a variety of African vipers and elapids as immunogens. As anticipated, both EAVs outperformed SAIMR polyvalent, with only EAV 1 proving inferior against the lethal effects of *E*. *carinatus* venom, while EAV 2 was only outperformed in experiments using *E*. *ocellatus* and *B*. *arietans* venoms (Fig 5).

## Discussion

There are a number of major obstacles that need to be tackled to ensure the effective treatment of tropical snakebite victims. These include, but are not limited to, addressing the poor affordability, lack of availability and the often limited cross-species neutralising potency of commercial antivenom [19,50]. Currently, all existing antivenoms are manufactured for specific geographical regions, typically parts of a continent, or specific countries within a continent, leading to a highly fragmented drug market [3,50]. Ultimately, the venoms used as immunogens for antivenom production dictate the breadth of snake species efficacy of those products, and it has been suggested that simply adding additional venoms to the immunogen mixture to increase paraspecific efficacy could be potentially counter-productive by diluting those antibodies specific to the toxins delivered during the bite by any particular snake [11,19,32]. Developing ‘generic’ or worldwide-applicable therapies for snakebite are ultimately confounded by the complexity in snake venom protein composition, with distinct toxins and therefore antigenicities found to vary from one species to another, and even within a single snake species as a consequence of ontogenetic and geographic variation [42,51].

Despite the challenge posed by venom variation, in the current pilot study we explored the feasibility of generating a globally efficacious conventional antivenom against haemotoxic envenoming, based on encouraging recent findings [42,52]. To do so, we used venom from a variety of geographically and taxonomically diverse and/or medically important haemotoxic snake species as immunogens. To explore the potential trade-off between antivenom dose efficacy and the number of venoms included in the immunising mixture, we generated two different EAVs, each consisting of ovine polyclonal antibodies, but using either seven or twelve different venoms in the immunising mixture. Time course analysis of the immunological responses of the sheep over the duration of immunisation (42 weeks), determined by ELISA analysis of serum samples, demonstrated highly comparable responses to the different immunogen mixtures, and high titres of cross-reactive antibodies resulting by the end of the immunising period. Thereafter, IgG was purified from each 42-week sample for use in in depth immunological and venom neutralisation experiments.

Despite considerable differences in the two different immunogen mixtures, ELISA and immunoblotting experiments revealed that both EAVs exhibited high levels of cross-reactivity and detected the vast majority of the variable protein constituents found in each of the 12 snake venoms (Figs 2 and 3A). The most noticeable exceptions to this were (i) disparities in the general intensity of immunological cross-reactivity and (ii) differences in the recognition of lower molecular weight toxins (i.e. <25 kDa) found in the immunoblotting experiments with the venoms of the non-front fanged snakes *D*. *typus* and *R*. *subminiatus*. EAV 2 outperformed EAV 1 on both counts, despite *D*. *typus* venom being included in both immunising mixtures, and these findings correlated with the binding levels observed in the end point ELISA experiments. These findings may be the result of the poor immunogenicity of venom components with low molecular weight [53–55], and/or due to distinctions between these non-viper venom components resulting in deficiency of these antigens to stimulate sufficient specific antibodies for experimental detection [56–58]. Contrastingly, the medium to high molecular weight toxins found across all of the various venoms (e.g. >25 kDa) were readily detected by both EAVs; including EAV 1 extensively recognising the majority of venom proteins found in the other venoms (i.e. the viperid venoms) not used as venom immunogens.

The other major and, perhaps more surprising, observation from the immunological assays was the seemingly low levels of antibody binding, coupled with reduced binding avidities, of both EAVs against *C*. *rhodostoma* venom (Fig 3). This was despite this venom being used as an immunogen to generate both antivenoms, and there being clear immunological cross-reactivity observed in the immunoblotting experiments (Fig 2). Notwithstanding these immunological observations, *in vitro* neutralisation assays revealed that both EAVs inhibited *C*. *rhodostoma* SVMP and coagulopathic venom activities in a manner highly comparable to the other venoms tested (Fig 4D and 4F). Indeed, broadly speaking, both antivenoms exhibited high levels of SVMP-inhibition against each of the venoms tested, and comparable findings were observed in terms of coagulotoxin inhibition, with the exception that EAV 2 provided little neutralisation effect against *E*. *ocellatus* venom at the dose tested, while neither antivenom inhibited *C*. *atrox* venom-induced coagulation. The inhibitory effects of both antivenoms against SVSP toxin activity was far more variable (Fig 4B), suggesting that both the titre and specificity of the resulting antibodies generated against these toxins may differ considerably between the two antivenoms, despite them sharing a number of snake venoms as immunogens.

One of the major challenges with interpreting the results described above in terms of predicted antivenom efficacy is that no single *in vitro* experiment accurately recapitulates the complex interaction of variable toxins on multiple physiological pathways following a snakebite. Indeed, some previous studies have demonstrated that high levels of immunological binding do not necessary result in effective neutralisation of venom toxicity *in vivo* [24,32]. Thus, to preliminarily assess the relative preclinical efficacies of the two EAVs described here, we employed an ethically-refined version of a previous described murine model of envenoming [49] and, to robustly investigate paraspecific neutralising capabilities, we used a diverse array of challenge venoms, including two that were not used as venom immunogens for generating either antivenom. Despite highly comparable immunological and *in vitro* inhibitory characteristics, our *in vivo* studies demonstrated that EAV 1 exhibited increased *in vivo* preclinical efficacy than EAV 2, despite fewer venoms being used in the immunogen mixture. Overall, at the single antivenom dose tested (5 mg [100 μl, 50 mg/ml]), EAV 1 completely protected experimental animals from the lethal venom effects of five of the eight diverse snake venoms tested, including two not used as immunogens (*B*. *arietans* and *V*. *ammodytes*) (Fig 5). In addition, EAV 1 provided some protection against lethality by *C*. *rhodostoma* and *E*. *carinatus* venoms. The former finding seems likely to reflect the low immunological binding observed against this venom, despite it being an immunogen, while the latter finding is more promising since this venom was not a venom immunogen, yet four of five experimental animals were protected in a paraspecific manner. EAV 1 was, however, completely ineffective at protecting against the effects of *L*. *muta* venom, a venom that was not included in either immunising mixture. These findings, which were also observed for EAV 2, suggest that *L*. *muta* venom contains lethal toxins sufficiently distinct from, or in much greater abundances than, those found in the venoms used as immunogens to prevent any degree of paraspecific neutralisation.

Our *in vivo* findings revealed that EAV 2 failed to provide complete protection against venom-induced lethality stimulated by any of the six venoms used as immunogens at the therapeutic doses tested (Fig 5). A degree of protection was observed against five of these six venoms (i.e. some experimental animals survived to the end of experiment), suggesting that the dose efficacy of EAV 2 is lower than that of EAV 1, and that perhaps with increased antivenom doses full protection might have been conveyed. Crucially, these findings provide experimental evidence that increasing the number of venoms in an immunogen mixture could perhaps have detrimental effects on efficacy, likely by diluting the proportional amount of venom neutralising antibodies specific to any particular venom via the addition of numerous immunogens (i.e. those venom toxins unique to the additional venoms used for immunisation). However, it should be stressed that our *in vitro* immunological experiments did not predict reductions in antibody binding to the various crude venoms, and thus more detailed follow up experiments involving purified toxins or chromatographically separated toxin fractions (i.e. antivenomic-type approaches [58,59]) are ultimately required to better understand the basis for these efficacy observations. Nonetheless, we suggest that a core set of venoms representing broad toxin diversity may be sufficient for the generation of antibodies that cross-react with and neutralise venoms in a paraspecific manner. Experimentally identifying the optimal venom mixture for immunisation remains challenging though, particularly since using too few venoms seems likely to result in reduced paraspecific efficacy, whereas too many may cause reductions in dose efficacy, as observed here.

Despite these findings, there are a number of limitations with the described work. For example, due to financial constraints, only a single animal was immunised to generate each EAV, and thus differences between the efficacy of the two antivenoms could be partially due to the different immune responses of these animals during immunisation. However, the highly comparable serological responses observed during the immunisation time course (Fig 1), and the comparable immunological cross-reactivity observed in ELISA and western blotting experiments (Figs 2 and 3), suggest that this is unlikely to be a major confounder of this study, though future iterations of attempts to generate pathology-focused experimental antivenoms should, where feasible, increase the number of immunisation animals to offset this risk. Another limitation relates to the restricted preclinical efficacy testing performed, which was a result of both ethical and financial constraints imposed on the study. Unlike the described *in vitro* neutralisation experiments, murine *in vivo* neutralisation experiments only utilised a subset of the venoms used as immunogens, and we only tested the generated EAVs at a single, relatively high, experimental therapeutic dose (5 mg [100 μl, 50 mg/ml]). Furthermore, animal monitoring of acute envenoming was only undertaken for 6 hours due to ethical considerations. Additional preclinical testing, involving those venoms omitted from this pilot work, and using varying antivenom doses to generate median effective dose data [48] for each venom, would provide a more complete picture of the efficacy and limitations of each EAV. However, due to evidence of incomplete neutralisation observed in these pilot studies, and the apparent need to further optimise the immunisation process to increase the paraspecific efficacy of these pathology-specific antivenoms, such additional experiments in more robust preclinical efficacy models [60] cannot be justified at this time.

Despite these limitations, this pilot study provides at least some evidence that generating anti-haemotoxicity antivenoms with global efficacy may be a viable future strategy for tackling snakebite envenoming, analogous with recent findings exploring the potential of pan-continentally efficacious anti-neurotoxicity antivenoms [41,42]. Careful selection of the specific venoms and/or venom toxins used as immunogens is of fundamental importance for stimulating adequate breadth of neutralisation against diverse snake species without compromising antivenom potency. Should further optimisation work address these current shortcomings, pathology-specific antivenoms may offer antivenom manufacturers with increased economies of scale to produce more sustainable conventional polyspecific antivenom products in the short to medium term to address the current therapeutic vacuum relating to tropical snakebite.

## Supporting information

S1 Fig End point titration ELISA analyses of immunological binding between the two experimental antivenoms (EAVs) and each of the individual venoms used as immunogens. The EAVs (EAV 1 and EAV 2), commercial SAIMR polyvalent antivenom (positive control) and normal sheep IgG and normal horse IgG (negative controls) were serially diluted fivefold and tested by ELISA against each of the haemotoxic venoms used as immunogens. Venoms from *B*. *asper*, *B*. *jararaca*, *E*. *ocellatus*, *C*. *rhodostoma*, *D*. *typus*, *D*. *acutus* and *D*. *russelii* were used as immunogens for EAV 1, while all of the venoms shown were used as immunogens for EAV 2. Error bars represent standard deviation (SD) of duplicate measurements.
(TIF)

S2 Fig *In vitro* immunological and neutralisation results for EAVs against each of the venom immunogen mixtures. **A)** End-point titration ELISA analysis of immunological binding between the EAVs and the two venom immunogen mixtures. All antibodies were serially diluted fivefold with a standardised starting concentration of 50 mg/ml. **B)** Chaotropic ELISA showing the relative avidity of the EAVs to the two venom immunogen mixtures in the presence of increasing molarities of the chaotropic agent ammonium thiocyanate (NH_4_SCN). The antibodies were used at a standardised concentration of 70 mg/ml. For both panels A and B, data points represent the mean of duplicate readings and error bars represent standard deviations. **C)** Quantification of the neutralisation of snake venom serine protease (SVSP) activity measured via chromogenic assay. Data points represent the rate of substrate cleavage measured kinetically. **D)** Quantification of the neutralisation of snake venom metalloproteinase (SVMP) activity measured via fluorescent assay. Data points represent area under the curve (AUC) of kinetic measurements. **E)** Quantification of the neutralisation of coagulopathic venom toxins via absorbance-based kinetic plasma clotting assay. The dashed line represents ‘normal clotting’ (i.e. negative control), with readings above the line promoting clotting (i.e. procoagulant), and those below inhibiting clotting (i.e. anticoagulant). The data represents percentage areas under the curve of the venom-only control area under the curve. For **(C)**, **(D)** and **(E)** the data points represent the mean of triplicate readings and error bars represent SEM. Throughout, the antivenoms used consisted of the two EAVs (EAV 1 and EAV 2), the SAIMR polyvalent antivenom control (positive control), and for the immunological assays, normal sheep and horse IgG were used as negative controls. PBS was used as the negative control in the functional assays, and *Bitis arietans* and *Echis ocellatus* venom were used as the positive controls in the serine protease and metalloproteinase assays, respectively.
(TIF)

S3 Fig The relative avidity of the EAVs to the venoms used as immunogens measured by immunological binding in the presence of increasing molarities of the chaotropic agent ammonium thiocyanate (NH_4_SCN). EAVs (EAV 1 and EAV 2), commercial SAIMR polyvalent antivenom (positive control) and normal sheep IgG and normal horse IgG (negative controls) (all at 1:10,000 dilutions) were tested in the presence of increasing concentrations of ammonium thiocyanate (0-8M). Venoms from *B*. *asper*, *B*. *jararaca*, *E*. *ocellatus*, *C*. *rhodostoma*, *D*. *typus*, *D*. *acutus* and *D*. *russelii* were used as immunogens for EAV 1, while all of the venoms shown were used as immunogens for EAV 2. Error bars represent SD of duplicate measurements.
(TIF)

S4 Fig The serine protease activity of the individual venom immunogens and their inhibition by the EAVs as measured by kinetic chromogenic assay. The SVSP venom activity (1 μg) of each individual venom immunogen is displayed as the rate of substrate conversion (kinetic readings at 405 nm over 30 mins). The antivenoms used consisted of the two EAVs (EAV 1 and EAV 2) and the commercial SAIMR polyvalent antivenom as an antivenom comparator, and PBS was used as the negative control. The positive control used across all experiments was *Bitis arietans* venom. Data points represent means of triplicate calculated rates, and error bars represent standard error of the mean (SEM).
(TIF)

S5 Fig The metalloproteinase activity of the individual venom immunogens and their inhibition by the EAVs as measured by kinetic fluorescent assay. The SVMP venom activity (1 μg) of each individual venom immunogen is displayed as area under the kinetic curve (AUC) of fluorescence (320 nm excitation and 405 nm emission over 40 mins). The antivenoms used consisted of the two EAVs (EAV 1 and EAV 2) and the SAIMR polyvalent antivenom as an antivenom comparator, and PBS was used as the negative control. The positive control used across all experiments was *Echis ocellatus* venom. The data displayed represents the mean AUC of triplicate measurements and error bars represent standard error of the mean (SEM).
(TIF)

S6 Fig The coagulopathic activity of the individual venom immunogens and their inhibition by the EAVs as measured by plasma coagulation assay. The data displayed shows the percentage of plasma clotting (normalised to venom only readings) of the means of triplicate area under the clotting curve measurements (absorbance plotted over time), with error bars represent the standard error of the mean (SEM). The dashed line represents normal clotting (i.e. negative control [PBS] readings). The antivenoms used consisted of the two EAVs (EAV 1 and EAV 2) and the SAIMR polyvalent antivenom as an antivenom comparator.
(TIF)

S1 Data A multi-tabbed excel file containing the raw data presented in the various figures of the manuscript. (XLSX)
