# Correction: Pathology-specific experimental antivenoms for haemotoxic snakebite: The impact of immunogen diversity on the *in vitro* cross-reactivity and *in vivo* neutralisation of geographically diverse snake venoms

**DOI:** 10.1371/journal.pntd.0010511

**Published:** 2022-06-02

**Authors:** Nessrin Alomran, Jaffer Alsolaiss, Laura-Oana Albulescu, Edouard Crittenden, Robert A. Harrison, Stuart Ainsworth, Nicholas R. Casewell

An error was identified in the S1 Data file arising from a copy and paste mistake. The file has now been updated to correct this error and correctly display the underpinning raw data.

## Supporting information

S1 Data A multi-tabbed excel file containing the raw data presented in the various figures of the manuscript. (XLSX)
