# Supplementary figures and images for: Pathology-specific experimental antivenoms for haemotoxic snakebite: The impact of immunogen diversity on the in vitro cross-reactivity and in vivo neutralisation of geographically diverse snake venoms
Source: PLoS Negl Trop Dis. 2021 Aug 18;15(8):e0009659. doi: 10.1371/journal.pntd.0009659 (PMC8423360; doi:10.1371/journal.pntd.0009659)

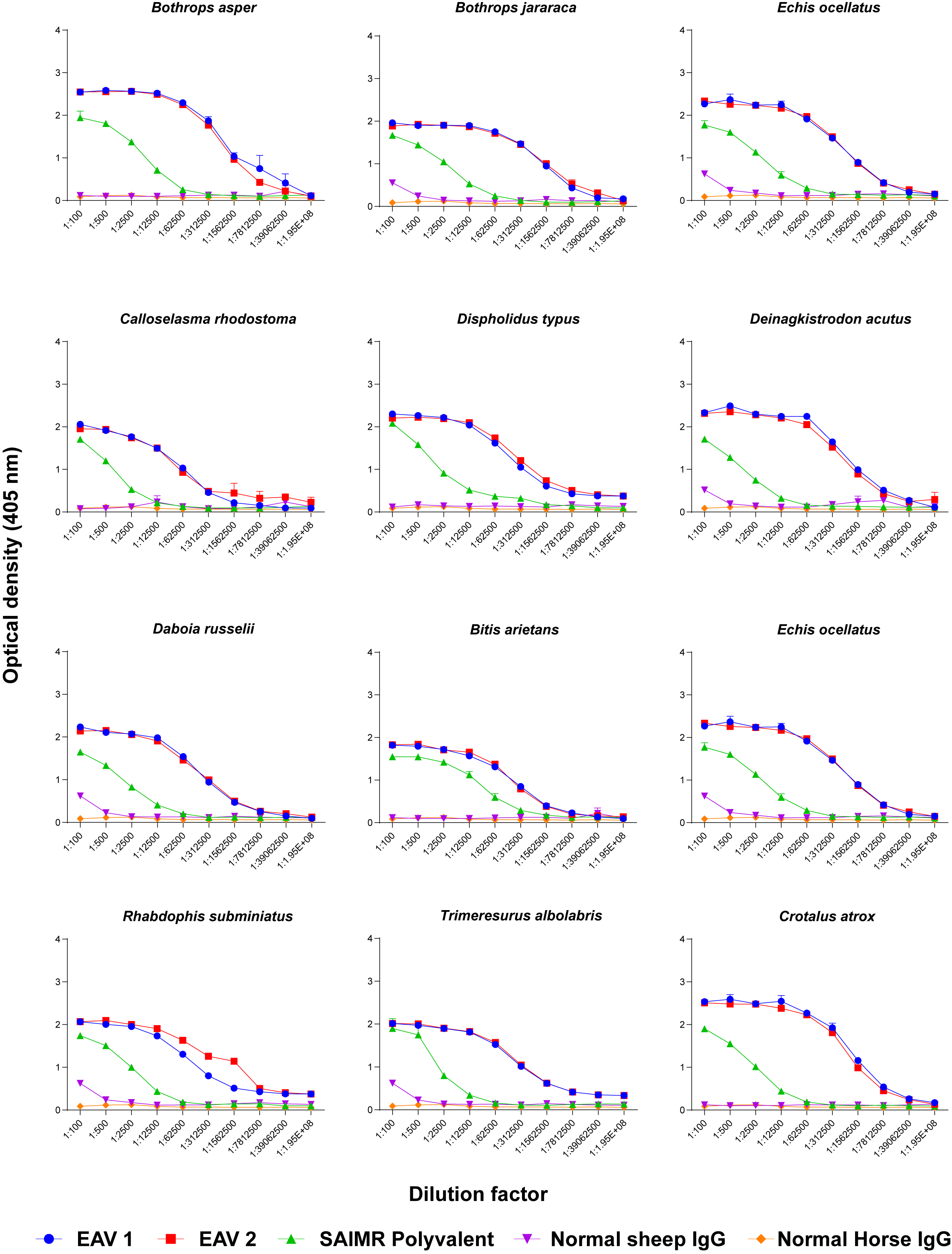

Supplement: S1 Fig — The EAVs (EAV 1 and EAV 2), commercial SAIMR polyvalent antivenom (positive control) and normal sheep IgG and normal horse IgG (negative controls) were serially diluted fivefold and tested by ELISA against each of the haemotoxic venoms used as immunogens. Venoms from B. asper, B. jararaca, E. ocellatus, C. rhodostoma, D. typus, D. acutus and D. russelii were used as immunogens for EAV 1, while all of the venoms shown were used as immunogens for EAV 2. Error bars represent standard deviation (SD) of duplicate measurements. (TIF) [file pntd.0009659.s001.tif]

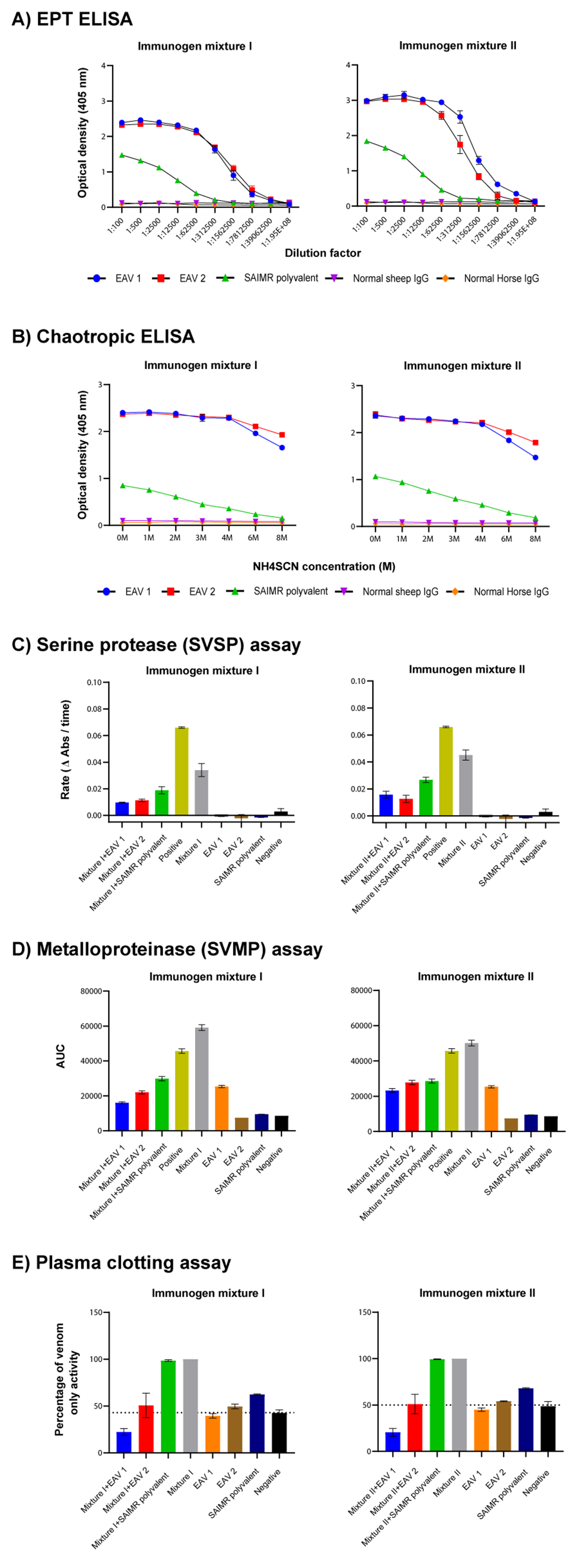

Supplement: S2 Fig — A) End-point titration ELISA analysis of immunological binding between the EAVs and the two venom immunogen mixtures. All antibodies were serially diluted fivefold with a standardised starting concentration of 50 mg/ml. B) Chaotropic ELISA showing the relative avidity of the EAVs to the two venom immunogen mixtures in the presence of increasing molarities of the chaotropic agent ammonium thiocyanate (NH4SCN). The antibodies were used at a standardised concentration of 70 mg/ml. For both panels A and B, data points represent the mean of duplicate readings and error bars represent standard deviations. C) Quantification of the neutralisation of snake venom serine protease (SVSP) activity measured via chromogenic assay. Data points represent the rate of substrate cleavage measured kinetically. D) Quantification of the neutralisation of snake venom metalloproteinase (SVMP) activity measured via fluorescent assay. Data points represent area under the curve (AUC) of kinetic measurements. E) Quantification of the neutralisation of coagulopathic venom toxins via absorbance-based kinetic plasma clotting assay. The dashed line represents ‘normal clotting’ (i.e. negative control), with readings above the line promoting clotting (i.e. procoagulant), and those below inhibiting clotting (i.e. anticoagulant). The data represents percentage areas under the curve of the venom-only control area under the curve. For (C), (D) and (E) the data points represent the mean of triplicate readings and error bars represent SEM. Throughout, the antivenoms used consisted of the two EAVs (EAV 1 and EAV 2), the SAIMR polyvalent antivenom control (positive control), and for the immunological assays, normal sheep and horse IgG were used as negative controls. PBS was used as the negative control in the functional assays, and Bitis arietans and Echis ocellatus venom were used as the positive controls in the serine protease and metalloproteinase assays, respectively. (TIF) [file pntd.0009659.s002.tif]

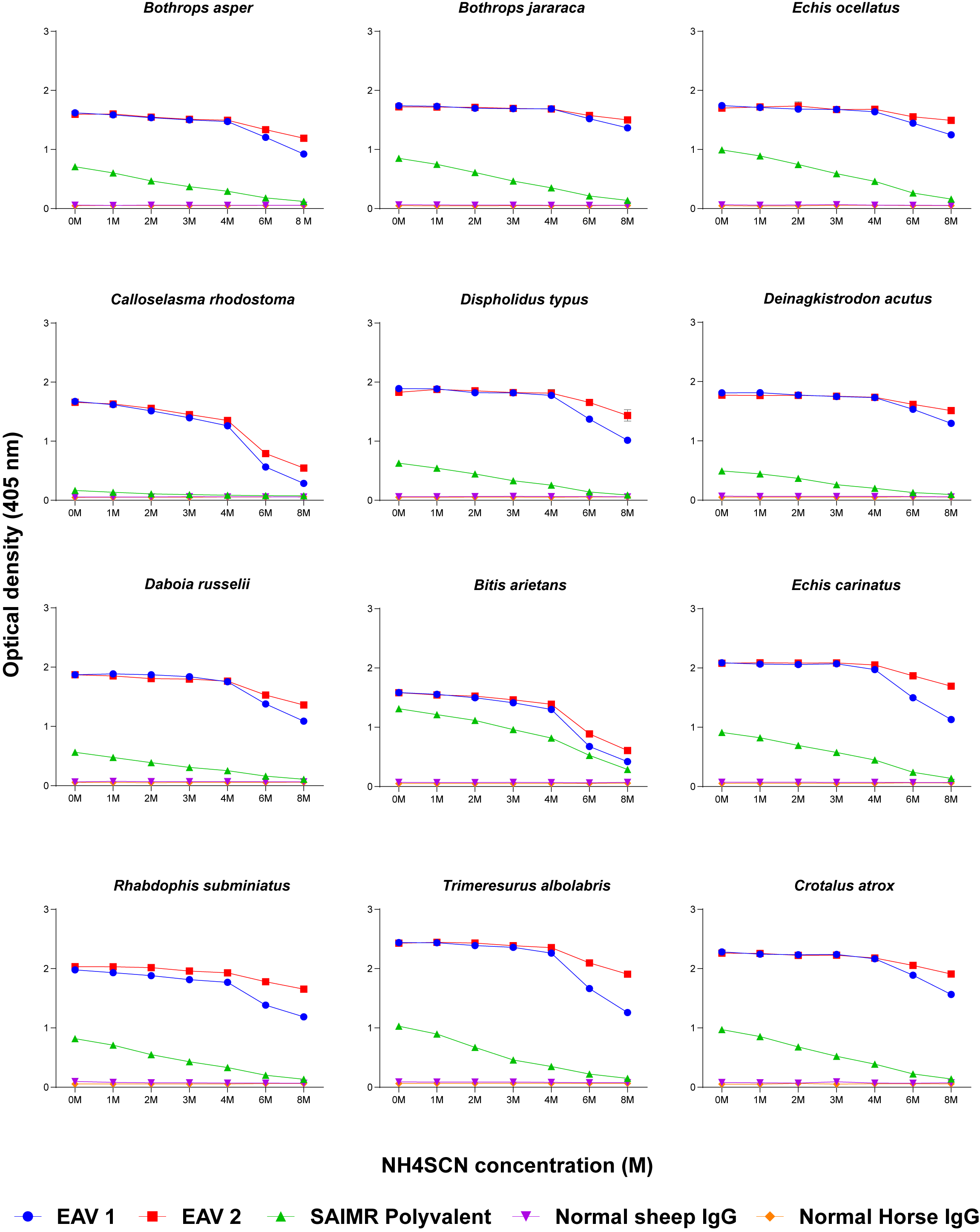

Supplement: S3 Fig — EAVs (EAV 1 and EAV 2), commercial SAIMR polyvalent antivenom (positive control) and normal sheep IgG and normal horse IgG (negative controls) (all at 1:10,000 dilutions) were tested in the presence of increasing concentrations of ammonium thiocyanate (0-8M). Venoms from B. asper, B. jararaca, E. ocellatus, C. rhodostoma, D. typus, D. acutus and D. russelii were used as immunogens for EAV 1, while all of the venoms shown were used as immunogens for EAV 2. Error bars represent SD of duplicate measurements. (TIF) [file pntd.0009659.s003.tif]

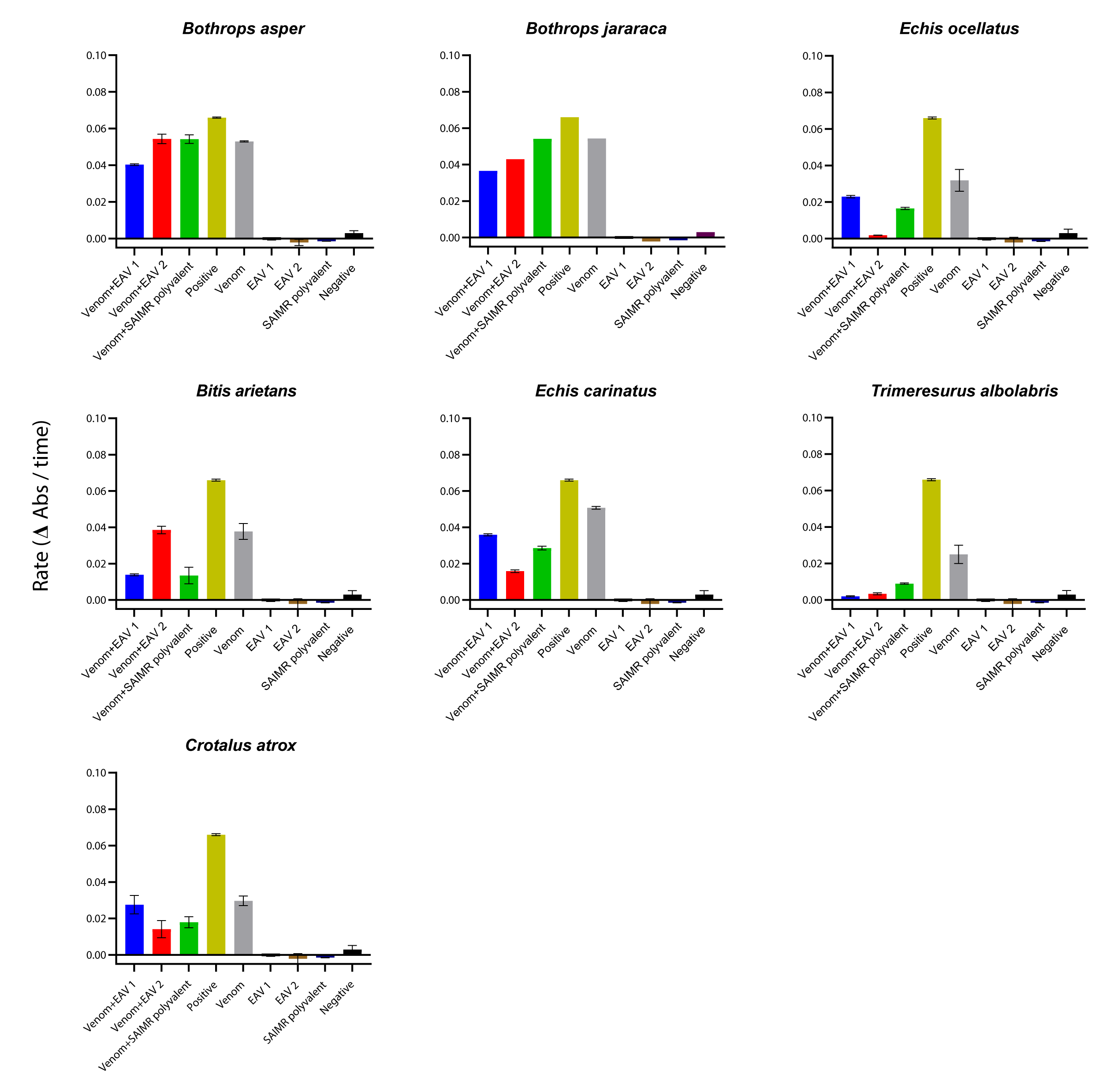

Supplement: S4 Fig — The SVSP venom activity (1 μg) of each individual venom immunogen is displayed as the rate of substrate conversion (kinetic readings at 405 nm over 30 mins). The antivenoms used consisted of the two EAVs (EAV 1 and EAV 2) and the commercial SAIMR polyvalent antivenom as an antivenom comparator, and PBS was used as the negative control. The positive control used across all experiments was Bitis arietans venom. Data points represent means of triplicate calculated rates, and error bars represent standard error of the mean (SEM). (TIF) [file pntd.0009659.s004.tif]

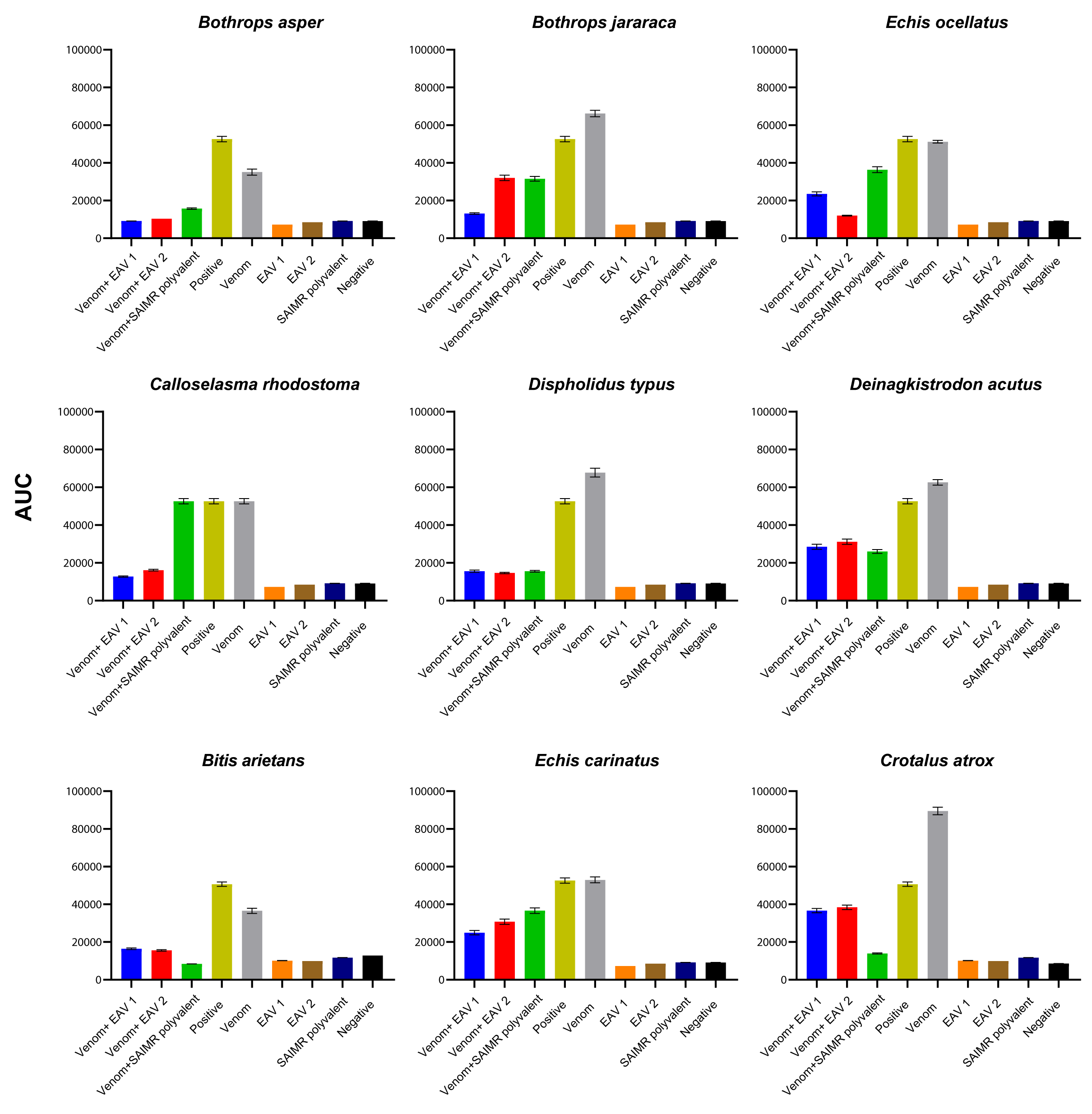

Supplement: S5 Fig — The SVMP venom activity (1 μg) of each individual venom immunogen is displayed as area under the kinetic curve (AUC) of fluorescence (320 nm excitation and 405 nm emission over 40 mins). The antivenoms used consisted of the two EAVs (EAV 1 and EAV 2) and the SAIMR polyvalent antivenom as an antivenom comparator, and PBS was used as the negative control. The positive control used across all experiments was Echis ocellatus venom. The data displayed represents the mean AUC of triplicate measurements and error bars represent standard error of the mean (SEM). (TIF) [file pntd.0009659.s005.tif]

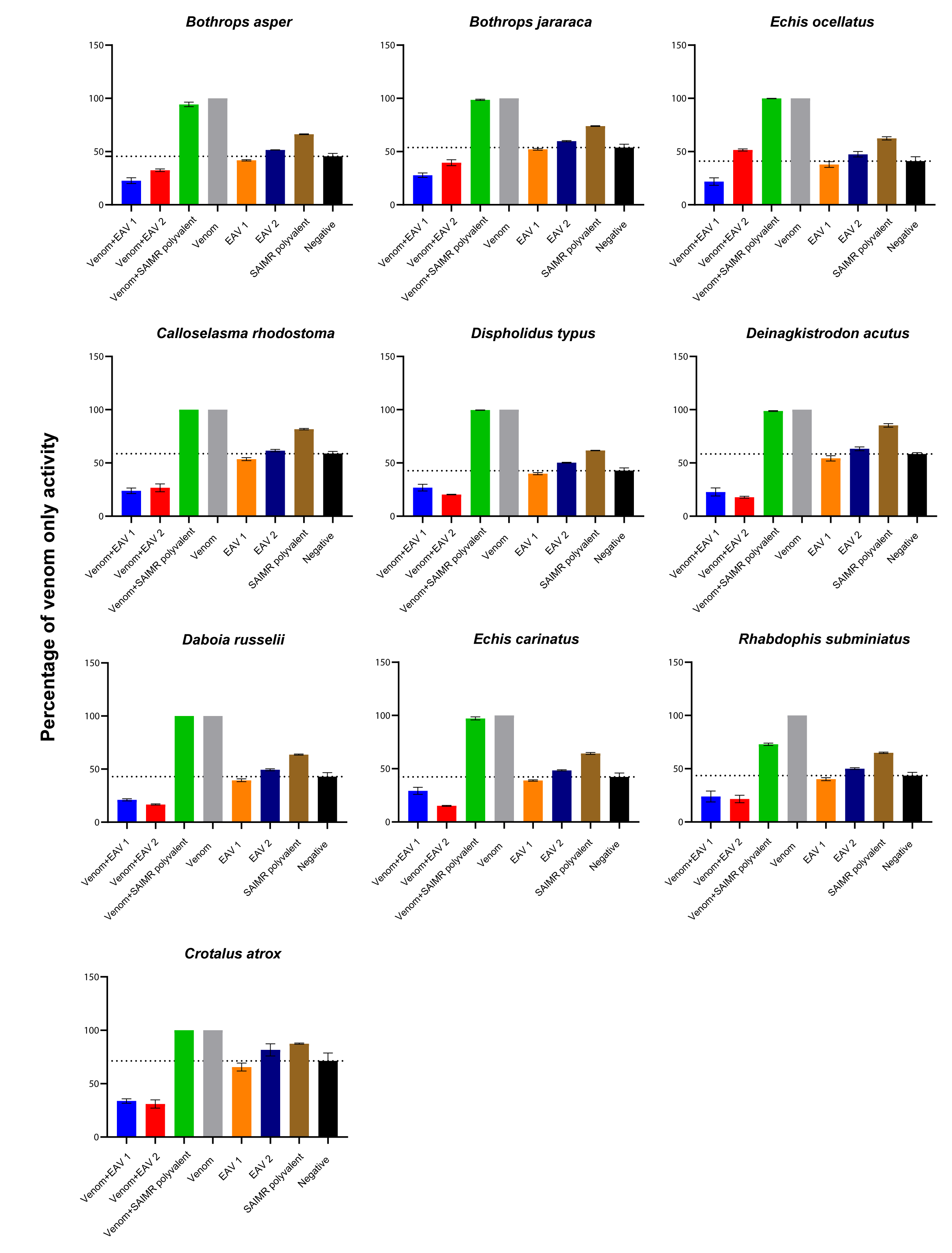

Supplement: S6 Fig — The data displayed shows the percentage of plasma clotting (normalised to venom only readings) of the means of triplicate area under the clotting curve measurements (absorbance plotted over time), with error bars represent the standard error of the mean (SEM). The dashed line represents normal clotting (i.e. negative control [PBS] readings). The antivenoms used consisted of the two EAVs (EAV 1 and EAV 2) and the SAIMR polyvalent antivenom as an antivenom comparator. (TIF) [file pntd.0009659.s006.tif]
